# Downregulation of circulating miR 802‐5p and miR 194‐5p and upregulation of brain MEF2C along breast cancer brain metastasization

**DOI:** 10.1002/1878-0261.12632

**Published:** 2020-02-05

**Authors:** Marta Sereno, János Haskó, Kinga Molnár, Sarah J. Medina, Zita Reisz, Rui Malhó, Mafalda Videira, László Tiszlavicz, Stephanie A. Booth, Imola Wilhelm, István A. Krizbai, Maria Alexandra Brito

**Affiliations:** ^1^ Faculdade de Farmácia Research Institute for Medicines (iMed.ULisboa) Universidade de Lisboa Portugal; ^2^ Institute of Biophysics Biological Research Centre Szeged Hungary; ^3^ Prion Diseases Section Public Health Agency of Canada National Microbiology Laboratory Winnipeg MB Canada; ^4^ Department of Pathology University of Szeged Hungary; ^5^ Faculdade de Ciências BioISI Instituto de Biossistemas e Ciências Integrativas Universidade de Lisboa Portugal; ^6^ Department of Galenic Pharmacy and Pharmaceutical Technology Faculdade de Farmácia Universidade de Lisboa Portugal; ^7^ Department of Medical Microbiology and Infectious Diseases Faculty of Health Sciences University of Manitoba Winnipeg MB Canada; ^8^ Institute of Life Sciences Vasile Goldiş Western University of Arad Romania; ^9^ Department of Biochemistry and Human Biology Faculdade de Farmmácia Universidade de Lisboa Portugal

**Keywords:** bioinformatics, brain metastasis, breast cancer, microRNAs, myocyte enhancer factor 2C, next‐generation sequencing

## Abstract

Breast cancer brain metastases (BCBMs) have been underinvestigated despite their high incidence and poor outcome. MicroRNAs (miRNAs), and particularly circulating miRNAs, regulate multiple cellular functions, and their deregulation has been reported in different types of cancer and metastasis. However, their signature in plasma along brain metastasis development and their relevant targets remain undetermined. Here, we used a mouse model of BCBM and next‐generation sequencing (NGS) to establish the alterations in circulating miRNAs during brain metastasis formation and development. We further performed bioinformatics analysis to identify their targets with relevance in the metastatic process. We additionally analyzed human resected brain metastasis samples of breast cancer patients for target expression validation. Breast cancer cells were injected in the carotid artery of mice to preferentially induce metastasis in the brain, and samples were collected at different timepoints (5 h, 3, 7, and 10 days) to follow metastasis development in the brain and in peripheral organs. Metastases were detected from 7 days onwards, mainly in the brain. NGS revealed a deregulation of circulating miRNA profile during BCBM progression, rising from 18% at 3 days to 30% at 10 days following malignant cells’ injection. Work was focused on those altered prior to metastasis detection, among which were miR‐802‐5p and miR‐194‐5p, whose downregulation was validated by qPCR. Using targetscan and diana tools, the transcription factor myocyte enhancer factor 2C (MEF2C) was identified as a target for both miRNAs, and its expression was increasingly observed in malignant cells along brain metastasis development. Its upregulation was also observed in peritumoral astrocytes pointing to a role of MEF2C in the crosstalk between tumor cells and astrocytes. MEF2C expression was also observed in human BCBM, validating the observation in mouse. Collectively, downregulation of circulating miR‐802‐5p and miR‐194‐5p appears as a precocious event in BCBM and MEF2C emerges as a new player in brain metastasis development.

## Graphical abstract

The search for aberrantly expressed miRNAs in plasma along breast cancer brain metastasis (BCBM) development revealed miR‐802‐5p and miR‐194‐5p downregulation as an early event. MEF2C emerged as their relevant target, with an increasing expression along BCBM expansion and in resected brain metastasis from breast cancer patients. Such miRNAs and MEF2C appear as early events and new players in BCBM development.

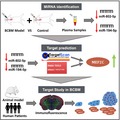

**Abbreviations**
3′UTR: 3′‐untranslated region
BBB: blood–brain barrier
BC: breast cancer
BCBM: breast cancer brain metastasis
BCCs: breast cancer cells
EDTA: ethylenediaminetetraacetic acid
GFAP: glial fibrillary acidic protein
MC: monoclonal
MEF2C: myocyte enhancer factor 2C
miRNAs or miR‐: microRNAs
NGS: next‐generation sequencing
PBS: phosphate‐buffered saline
PC: polyclonal
qPCR: quantitative real‐time polymerase chain reaction

## 1 Introduction

Breast cancer (BC) is the most frequently diagnosed cancer among women worldwide. It represents about 12% of all new cancer cases and 25% of cancers in women, being one of the leading causes of female death from cancer, according to the International Agency for Research on Cancer (Ferlay *et al.*, 2015; Torre *et al.*, 2016). BC has a high incidence and is, next to lung cancer, the second most frequent cause of brain metastases, which are usually associated with a poor prognosis and diminished life expectancy after diagnosis (Leone *et al.*, 2015). After transmigrating to the brain, BC cells (BCCs) encounter an ideal environment for metastatic growth, since the blood–brain barrier (BBB) restricts the entrance of most chemotherapeutic agents and provides protection against immune surveillance (Winkler, 2015). Moreover, the BBB and the crosstalk between malignant and brain cells favor brain metastasis (Wilhelm *et al.*, 2019). Altogether, this renders the brain a sanctuary against antitumor strategies, hindering the treatment of brain metastases with the available current therapies (Kotecki *et al.*, 2018).

MicroRNAs (miRNAs or miR‐) are a subclass of single‐stranded small noncoding RNAs with about 21–25 nucleotides long that are endogenously produced and found in diverse organisms, including humans. They play important gene‐regulatory roles by pairing to the 3′‐untranslated region (3′UTR) of mRNAs of protein‐coding genes to direct their posttranscriptional regulation, mainly by repressing their expression (Wahid *et al.*, 2010). For any given miRNA, there are a large number of potential target sites, even within the same gene, with a number of factors influencing miRNA‐mRNA interactions (Lim *et al.*, 2005). Recently, both tissue and circulating miRNAs have arisen as efficient and specific biomarkers for different types of cancer and metastases, having specific expression profiles (He *et al.*, 2015). Particularly, circulating miRNAs can be of interest, due to their high stability and easy quantification in biofluids (Chen *et al.*, 2008), being key components of liquid biopsies, which are more and more replacing traditional biopsies for diagnosis and prognosis of cancer (Karachaliou *et al.*, 2015).

Despite the emerging role of miRNAs in different types of cancers and metastases, data about their expression pattern in BC brain metastasis (BCBM) are limited. This prompted us to profile miRNAs along BCBM development, identify their target genes, assess the corresponding protein expression in the mouse brain, and further validate their presence in resected brain metastasis samples from BC patients.

## 2 Materials and methods

### 2.1 Cell culture and mouse model of breast cancer brain metastasis

A mouse model of BCBM, relying on the inoculation of murine mammary carcinoma triple‐negative 4T1 cells in the carotid artery of Balb/c mice, was used. 4T1 cells, purchased from ATCC (Middlesex, UK), were maintained in RPMI 1640 Medium (PAN Biotech, Aidenbach, Germany) supplemented with ultraglutamine I (Lonza, Basel, Switzerland) and 5% heat‐inactivated fetal bovine serum (PAN Biotech) in a 5% CO_2_ atmosphere at 37 °C. These metastatic BCCs (1 × 10^6^ cells in a total volume of 200 µL of Ringer‐HEPES) were xenografted, under isoflurane anesthesia, in the right common carotid artery of 7‐ to 8‐week‐old female Balb/c mice, purchased from Charles River Laboratories (Wilmington, MA, USA). Control mice were inoculated with Ringer‐HEPES. Mice were housed and bred in the animal facility of the Biological Research Centre, Szeged, Hungary. Plasma samples were collected (*n* = 5), and brains, lungs, kidneys, and livers were harvested (*n* = 6), 5 h, 3 days, 7 days, or 10 days postinoculation.

All animal experimentation was performed by certified team members at the Biological Research Centre, according to the recommendations of the Declaration of Helsinki and Tokyo, and was performed according to the EU Directive 2010/63/EU on the protection of animals used for experimental and other scientific purposes. The protocol was reviewed and approved by the Regional Animal Health and Food Control Station of Csongrád County (license numbers: XVI./2980/2012 and XVI./764/2018).

#### 2.1.1 Plasma sample collection

Blood samples were collected for analysis of miRNA expression in plasma by next‐generation sequencing (NGS) and further validation by quantitative real‐time polymerase chain reaction (qPCR). Approximately 500 µL blood samples were collected directly from the heart of live mice under isoflurane anesthesia. The samples were collected using syringes previously washed with ethylenediaminetetraacetic acid (EDTA, 0.5 m, pH 8.0) into tubes containing 40 µL of the anticoagulant. After collection, the blood samples were centrifuged for 10 min at 380 ***g***, at 4 °C, and the plasma was collected. The plasma samples were stored at −80 °C until further analysis.

#### 2.1.2 Organ collection

For histological analysis of metastasis development in selected brain regions and in peripheral organs, brains, lungs, kidneys, and livers were harvested at the different timepoints after injection. Anesthetized mice were perfused with 50 mL of phosphate‐buffered saline (PBS), followed by 25 mL of 4% paraformaldehyde in PBS to fix the tissues. The brains, lungs, kidneys, and livers were harvested and postfixed overnight in 4% paraformaldehyde in PBS at 4 °C and afterward were kept in PBS containing 0.1% sodium azide. The cerebellum, cranial hippocampus, and striatum (coronal sections at −6.12, −1.82, and 0.5 mm Bregma coordinates, respectively), as well as lungs, kidneys, and livers, were paraffin‐embedded and serially cut into 4‐μm‐thick sections.

### 2.2 Human brain metastasis samples

Six‐micrometre‐thick paraffin‐embedded sections of resected human brain metastases were obtained from the Department of Pathology of the University of Szeged, Szeged, Hungary. Samples were collected from female patients (*n* = 4), with stage IV triple‐negative breast cancer with well‐established brain metastases. Human samples were collected in accordance with the permission of the Human Investigation Review Board, University of Szeged (Permit number: EMLOSEB001, project title: Retrospective analysis of surgical samples of breast cancer patients), issued on January 31, 2017, in agreement with the Declaration of Helsinki of the World Medical Association.

### 2.3 Histological and immunofluorescence analysis

#### 2.3.1 Hematoxylin–eosin staining

For hematoxylin–eosin staining, the tissue was deparaffinized in xylene (Klinipath, Duiven, Netherlands) (10 min), rehydrated in successive ethanol solutions (100% ethanol for 3 min, 96% ethanol for 3 min, and 70% ethanol for 3 min), and in tap water (1 min). The nuclei were stained with Papanicolaou’s solution 1a Harris’ hematoxylin solution (Merck Millipore, Darmstadt, Germany) for 10 min. Sections were then differentiated using a solution of 1% hydrochloric acid in 70% ethanol (20 s) and bluing in 1% ammonia water (10 s). The cytoplasm was stained with eosin Y solution 0.5% alcoholic (Merck Millipore) for 2 min. Finally, sections were dehydrated in a series of alcohols (70% ethanol for 3 min, 96% ethanol for 3 min, and 100% ethanol for 3 min) and diaphanized in xylol for 4 min and finally mounted with Quick‐D Mounting Medium (Klinipath).

Photographs of hematoxylin–eosin staining were acquired with an Olympus BX51 Microscope equipped with DP50 digital camera and Olympus Plan Apo objectives (Labocontrole, Lisbon, Portugal). For analysis of metastasis extension and distribution, the area of metastases in each brain region and peripheral organs was measured in 10 fields per organ or region, at each timepoint, using imagej 1.29x software (National Institutes of Health, USA). The results are presented as the ratio of tumor area to tissue area.

#### 2.3.2 Immunofluorescence

Brain sections were processed for immunofluorescence analysis of myocyte enhancer factor 2(MEF2)C, claudin‐5, pan‐cytokeratin, and glial fibrillary acidic protein (GFAP). The experimental conditions are described below and summarized in Table 1. Sections were deparaffinized in xylene (20 min) and rehydrated through successive immersion in 100% ethanol (20 min), 96% ethanol (10 min), 70% ethanol (10 min), and finally tap water (10 min). Heat‐mediated antigen retrieval was performed with 10 mm citrate buffer pH 6.0 during 15 min in the microwave. A permeabilization step was performed with 0.5% Triton X‐100 for 15 min, and tissue sections were blocked with the appropriate blocking solutions for 60 min. The primary antibodies were diluted in the respective blocking solutions with 0.5% Triton X‐100, and sections were incubated overnight at 4 °C. Then, the incubation with the respective fluorescently labeled secondary antibody diluted in the respective blocking solutions with 0.5% Triton X‐100 was performed during 60 min at room temperature. Between the several steps after the antigen retrieval treatment, washes with PBS (10 min) were performed. Negative controls with omission of primary antibodies were performed to exclude nonspecific binding or cross‐reactivity. Nuclei were labeled with Hoechst 33342 dye diluted 1 : 1000 in PBS for 10 min, followed by mounting with SlowFade® Diamond Antifade Mountant (both from Thermo Fisher Scientific, Waltham, MA, USA).

**Table 1:** Summary of the experimental conditions used for immunofluorescence analysis of brain tissue.

Marker	Blocking	Primary antibody	Dilution	Secondary antibody	Dilution
MEF2C	10% goat serum/3% BSA	Thermo Fisher Scientific, #PA5‐28247, Rabbit PC	1 : 100	Alexa Fluor® 555 goat anti‐rabbit Thermo Fisher Scientific, #A‐21235	1 : 250
Claudin‐5	10% goat serum	Thermo Fisher Scientific, #35‐2500, Mouse MC	1 : 250	Alexa Fluor® 647 goat anti‐mouse Thermo Fisher Scientific, #A‐21235	1 : 500
Pan‐cytokeratin	10% goat serum	Thermo Fisher Scientific, #MA5‐12231, Mouse MC	1 : 500	Alexa Fluor® 647 goat anti‐mouse Thermo Fisher Scientific, #A‐21235	1 : 500
GFAP	3% BSA	Sigma Aldrich. #G3893, Mouse MC	1 : 1000	Alexa Fluor® 488 goat anti‐mouse Thermo Fisher Scientific, #A‐11001	1 : 500

BSA, bovine serum albumin; GFAP, glial fibrillary acidic protein; MC, monoclonal; MEF2C, myocyte enhancer factor 2C; PC, polyclonal.

Thin optical images (c. 3 µm thick) were acquired using a Leica SP‐E confocal microscope (Leica Microsistemas, Lisbon, Portugal), equipped with 488, 532, and 635 nm lasers and operating in the mode 1024 × 1024, 400 Hz (c. 1/3 s per frame). For immunofluorescence analysis, ten fields of the cranial hippocampus (the brain region more prone to metastasis development) of each animal were acquired under the same conditions; MEF2C expression was analyzed based on the evaluation of total fluorescence by tumor area, determined by delimitation of each metastasis, using imagej software. Results were expressed as fluorescence intensity by μm^2^ of tumor area. For evaluation of MEF2C nuclear translocation in metastasis, the number of cells with nuclear expression in each metastasis was counted and compared with the total number of cells in each metastasis. The results were presented in percentage of cells with MEF2C nuclear expression.

### 2.4 miRNA analysis

#### 2.4.1 Next‐generation sequencing

For the NGS analysis, RNA was extracted from 50 to 200 µL of plasma using Norgen’s Plasma/Serum RNA Purification Mini Kit and eluted in 25 µL. Due to small amounts of RNA, samples from the same treatment were pooled and 48 ng of RNA was used for library preparation using Illumina’s TruSeq Small RNA Kit (Illumina, San Diego, CA, USA). Samples were indexed accordingly for multiplexing, and 15 cycles of PCR amplification were performed. To obtain the desired product size, a BluePippin isolation (Sage Science Inc., Beverly, MA, USA) using a 3% agarose gel and gated at 120–140 bp, followed with a clean‐up using AMPure XP beads, was performed. To confirm that the desired product was selected, the cleaned‐up library‐prepped samples were run on Agilent’s Bioanalyzer using a High Sensitivity DNA chip. These samples were run on a MiSeq v3 Reagent Kit flow cell from Illumina, performing 85 SR cycles. The resulting FASTQ files were uploaded into Genboree Workbench (genboree.org) where miRNA read counts were determined using the exceRpt small RNA‐seq Pipeline v4.6.2 and compared against mouse genome mm10 (Gene Expression Omnibus # http://www.ncbi.nlm.nih.gov/geo/query/acc.cgi?acc=GSE136149).

#### 2.4.2 RNA extraction, cDNA preparation, and qPCR

For the qPCR, we first isolated total RNA from the plasma samples using the miRCURY RNA Isolation Kit for biofluids (Exiqon, Vedbaek, Denmark), according to manufacturer’s instruction. RNA was then transcribed into cDNA, using the reverse transcription kit Universal cDNA Synthesis Kit II (Exiqon), according to manufacturer’s instructions for plasma. However, the initial RNA volume was increased four times in a final volume of reaction of 15 µL. Prior to the reverse transcription reaction, the synthetic RNA Spike‐in Uni‐SP6 (Exiqon) was added to the mixture. The reaction was performed on a Bio‐Rad iQ5 thermocycler, using the following conditions: 42 °C for 60 min; 95 °C for 5 min to heat‐inactivate the reverse transcriptase; and cooling down and storage at 4 °C. The qPCR was performed using the same equipment and miRCURY LNA SYBR Green PCR Kit (Exiqon) according to manufacturer’s instructions using cDNA diluted at 1 : 6. The following conditions were used: 50 cycles of 95 °C for 15 s, 56 °C for 30 s, 72 °C for 30 s, and a ramp rate of 1.6 °C·s^−1^, followed by a melting curve analysis. Predesigned LNA primer pairs were purchased from Exiqon for each of the selected miRNAs (mmu‐miR‐802‐5p, mmu‐miR‐194‐5p) and for miR‐16‐5p that was used as an endogenous control to normalize the expression level. qPCR was performed in 96‐well plates, with each sample performed in triplicate, and a no‐template control was included for each amplification. Determination of the threshold cycle was performed using the bio‐rad iq5 thermocycler’s software, while the quantitation was determined by the comparative Ct method. The results were presented as fold change (FC). For quality control, the PCR products were run by electrophoresis on a 4% agarose gel (1% Agarose + 3% NuSieve Agarose) for 1 h at 100 mV to confirm the presence and expected size of the PCR products.

### 2.5 Bioinformatical target prediction

To predict possible targets of each miRNA, a bioinformatical analysis was performed using two different target prediction tools, available online: targetscan v.7.2 (Agarwal *et al.*, 2015) and diana tools microt‐cds v.5.0. (MD *et al.*, 2013). targetscan categorizes miRNAs by the state of the families’ conservation. For diana tools microt‐cds, a threshold of 0.7 was applied as recommended by the software. The results of the target prediction were sorted by the scores obtained with targetscan (cumulative weighted context ++ score, total context score, and aggregate PCT) and with diana tools microT‐CDS (miTG). Total context score is the sum of the contribution of 14 features for each of the four seed site types, with the most negative total context score value representing the highest probability of repression. The cumulative weighted context score was calculated using total context scores and cumulative predicted repression at different sites existing in a target mRNA. It estimates the total repression expected from multiple sites of the same miRNA for each target. Values can be between −1 and 1, but the more negative the value, the greater the repression (Agarwal *et al.*, 2015). It is the most relevant score, regarding efficacy of repression prediction by targetscan, which also gives a probability of preferentially conserved targeting, the aggregate PCT. This score is an estimate of the probability that a site is conserved due to the maintenance of miRNA targeting, rather than by chance or any other reason not pertinent to miRNA targeting (Friedman *et al.*, 2009). Finally, the miTG score, calculated by diana tools, is a general score for the predicted interaction. The higher the score, the greater the confidence (Riffo‐Campos *et al.*, 2016). The results obtained with both tools were compared, and only the targets predicted by both tools were considered. Due to the high number of predicted targets for some miRNAs, a bibliographical search was done to select the targets with more relevance for further validation.

### 2.6 Statistical analysis

Results were analyzed using graphpad prism® 5.0 (GraphPad Software, San Diego, CA, USA). Immunofluorescence and hematoxylin–eosin results are expressed as mean ± SEM. A one‐way ANOVA, followed by the Bonferroni *post hoc* test, was used to evaluate whether there were statistically significant changes in parameters measured by immunofluorescence and hematoxylin–eosin, between the different timepoints and studied organs or brain regions. qPCR results are expressed as mean ± SD. A two‐tailed *t*‐test was used to evaluate whether there were significant changes in the expression of the different miRNAs in the 4T1 group, when comparing to the control group. *P* values < 0.05 were considered statistically significant.

## 3 Results

### 3.1 Well‐established metastases are detected in the brain from 7 days onwards after inoculation of breast cancer cells

**Figure 1:**
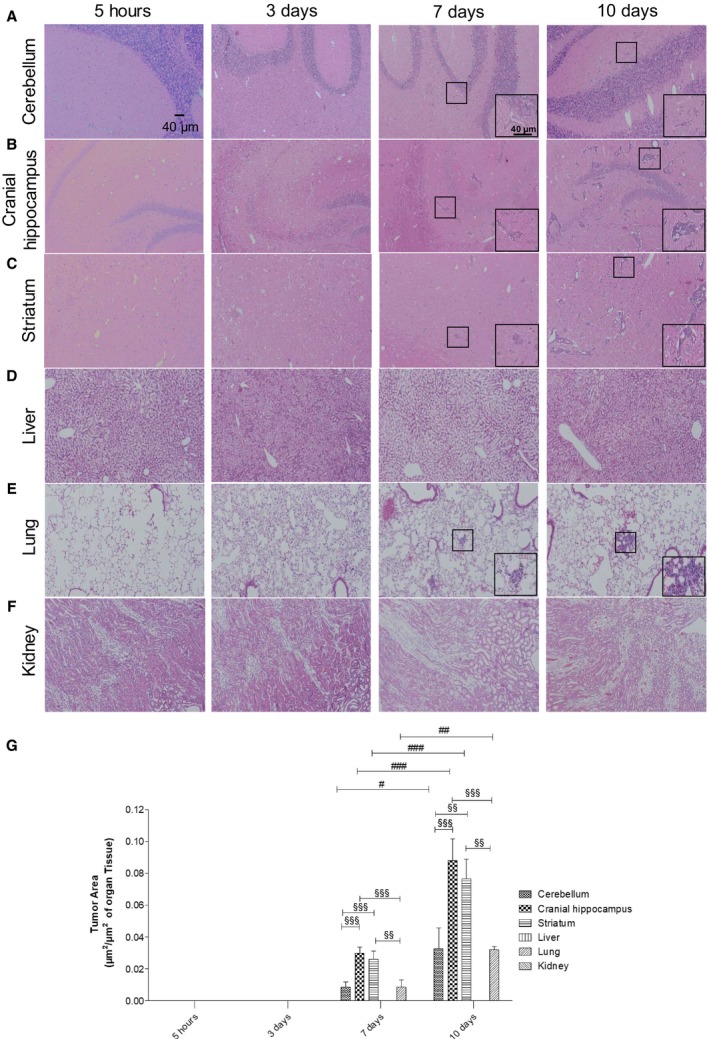
Profile of breast cancer metastases in the brain and peripheral organs. Hematoxylin–eosin staining of cerebellum (A), cranial hippocampus (B), striatum (C), liver (D), lung (E), and kidney (F) was performed, and the tumor area was quantified (G) at several timepoints after inoculation of triple‐negative breast cancer cells in 7‐ to 8‐week‐old female Balb/c mice (*n* = 6). Insets show the magnification of the selected representative metastasis (inside the squares). The results are expressed as mean ± SEM. A one‐way ANOVA, followed by the Bonferroni *post hoc* test, was used to evaluate the significant changes in parameters, between the different timepoints and studied organs. ^#^ *P* < 0.05, ^##^ *P* < 0.01, and ^###^ *P* < 0.001 between indicated timepoints, for the same regions; ^§§^ *P* < 0.01 and ^§§§^ *P* < 0.001 between indicated regions for the same timepoint.

To check the metastatic pattern in the used animal model, the tumor area was determined at different timepoints after injection of the tumor cells (5 h, 3, 7, and 10 days), in three brain regions (cerebellum, cranial hippocampus, and striatum), as well as in peripheral organs (lungs, kidney, and liver). Observation of hematoxylin–eosin‐stained brain sections revealed that at 5 h and 3 days, no metastases were detectable in any of the studied regions, whereas their presence was detected at 7 days and even more at 10 days (Fig. 1A–C). Regarding the peripheral organs, metastases were only detected in the lungs (Fig. 1D–F). Analysis of metastasis area revealed that the most affected brain region both at 7 and 10 days was the cranial hippocampus, immediately followed by the striatum, while the least affected one was the cerebellum (Fig. 1G). It also revealed that the tumoral area in the lungs was similar to that of the least affected brain region. These results show that 4T1 cells in this mouse model preferentially metastasize to the brain; therefore, this is a good *in vivo* model for the study of peripheral events associated with brain metastasization. Since the brain region with the highest tumoral area was the cranial hippocampus, this region was selected for the subsequent brain analyses.

### 3.2 miRNAs are aberrantly expressed in plasma of 4T1‐injected mice along the metastasization process

**Figure 2:**
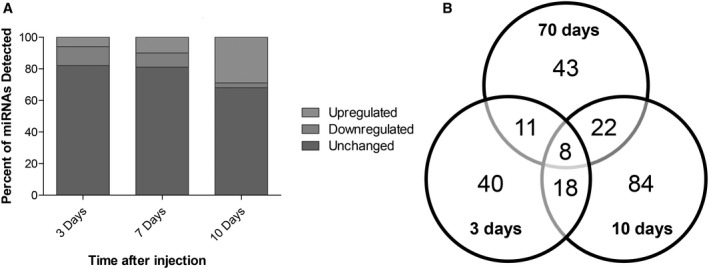
Overview of the next‐generation sequencing (NGS) results, regarding the altered expression of microRNAs (miRNAs) in plasma along brain metastasization of breast cancer. Plasma samples were collected at several timepoints after inoculation of triple‐negative breast cancer cells, or vehicle (control), in 7‐ to 8‐week‐old female Balb/c mice. NGS miRNA analysis was performed, followed by analysis of the number of miRNAs whose expression was altered in comparison with the corresponding timepoint control. miRNAs with fold change from control > 2.0 were considered to be significantly upregulated, while miRNAs with fold change < 0.5 were considered to be downregulated. The remaining miRNAs were considered unchanged. The percentage of upregulated, downregulated, or unchanged miRNAs was calculated for each timepoint (A). Venn diagram indicating the number of overlapping and nonoverlapping deregulated miRNAs in the three studied timepoints (B).

As previously mentioned, it has been suggested that different types of metastasis can have unique circulating miRNA signatures (Rosenfeld *et al.*, 2008). However, no specific miRNA profile has yet been described for BCBM. So, once the metastatic profile of this model was established, our aim was to identify the specific circulating miRNA signature of BCBM by finding aberrantly expressed miRNAs. To this end, plasma samples collected from 4T1‐injected mice and from controls at 3, 7, and 10 days were subjected to NGS analysis. To render feasible the analysis of the large data obtained from the NGS analysis, we established as arbitrary criteria the miRNAs that were upregulated or downregulated, with FC > 2.0 or FC < 0.5, respectively. Although most miRNAs remained unchanged for all the timepoints, the percentage of deregulated ones increased with time (Fig. 2A). While at 3 and 7 days ~ 18% of the miRNAs were deregulated, that percentage increased to ~ 30% at 10 days. An interesting observation within the deregulated miRNAs was that throughout time, the number of downregulated miRNAs decreased, while the number of upregulated ones increased. In fact, at 3 days 77 miRNAs were found to be altered (Fig. 2B), 52 of which were downregulated, while the remaining 25 were upregulated; so, more than half of the deregulated miRNAs were downregulated. At 7 days, 84 miRNAs were deregulated but approximately half of the miRNAs were upregulated (43 miRNAs). Contrarily, at 10 days, a total of 133 miRNAs were deregulated but only 11 of them were downregulated, while the remaining 122 were upregulated. So, besides having more deregulated miRNAs at 10 days, the number of upregulated miRNAs suffered a dramatic increase, comparing to 3 and 7 days. Moreover, comparison between the altered miRNAs among the different timepoints revealed that they were not all the same in all the timepoints. Indeed, while most miRNAs were solely deregulated at a single timepoint, only eight miRNAs were altered at 3, 7, and 10 days after injection of the 4T1 cells (Fig. 2B), namely miR‐34c‐3p, miR‐335‐3p, miR‐708‐3p, miR‐690, miR‐340‐3p, miR‐425‐3p, miR‐130a‐5p, and miR‐449a‐5p. Altogether, these results demonstrated that the miRNA signature varies throughout time and that those that are altered prior to metastasis are not necessarily the same that are deregulated in more advanced stages of the tumor. These results also showed that at more advanced stages of tumor progression, miRNAs tend to be more upregulated than in earlier stages.

### 3.3 A specific set of miRNAs is aberrantly expressed prior to detectable metastasis formation

**Figure 3:**
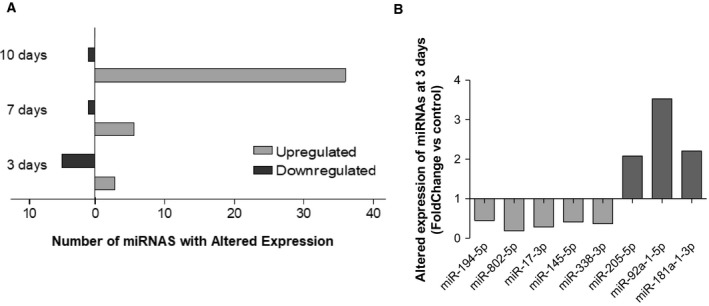
Up‐ and downregulated microRNAs (miRNAs) with read counts higher than 20 000, throughout metastatic progression and specific miRNAs that were altered prior to metastasis and selected for further studies. Next‐generation sequencing results were narrowed down based on their fold change from control and read counts, and only the miRNAs with read counts higher than 20 000 were considered for each timepoint (A). At the timepoint of interest for this study (3 days), eight miRNAs were identified, five of which were downregulated (fold change < 0.5), while three were upregulated (fold change > 2.0) (B).

Since the high number of altered miRNAs (Fig. 2B) precludes the validation of all of them, we selected for further analysis the miRNAs with the highest expression considering those with read counts higher than 20 000, either in the control samples or in 4T1‐injected samples. In these conditions, the number of aberrantly expressed miRNAs was 8, 7, and 39, at 3, 7, and 10 days, respectively (Fig. 3A). Considering the relevance that precociously altered miRNAs in plasma may have as potential biomarkers of brain metastasis development, we focused our studies in those altered at 3 days (prior to brain metastasis detection) and present the miRNAs that were deregulated at 7 and 10 days in Tables S1 and S2. Among the altered miRNAs at 3 days, five were downregulated (miR‐194‐5p, miR‐802‐5p, miR‐17‐3p, miR‐145‐5p, and miR‐338‐3p), while three were upregulated (miR‐205‐5p, miR‐92a‐1‐5p, and miR‐181a‐1‐3p), as indicated in Fig. 3B. None of these maintained a deregulated expression throughout the three timepoints, indicating that their aberrant expression is characteristic of early stages of BCBM progression.

### 3.4 Target prediction for the selected miRNAs, through a bioinformatical approach

Once aberrantly expressed miRNAs were identified, we further wanted to identify their targets with relevance for the development of BCBM. However, the high number of single miRNA targets and miRNA‐mRNA interactions, and the fact that they are influenced by numerous conditions, renders challenging their correct identification. Nowadays, numerous web‐based bioinformatical tools provide algorithms to predict potential miRNA target genes and interactions, which allows narrowing down the potential targets for experimental validation. So, a bioinformatical prediction of the targets for each of the miRNAs with an early aberrant expression (Fig. 3B) was performed using targetscan v.7.2 and diana tools microt‐cds v.5.0. The selected miRNAs were entered in both tools according to the following descriptives: mmu‐miR‐194‐5p, mmu‐miR‐802‐5p, miR‐17‐3p, mmu‐145‐5p, miR‐338‐3p, mmu‐miR‐205‐5p, mmu‐miR‐92a‐1‐5p, and mmu‐miR‐181a‐1‐3p. Conservation is an important aspect of miRNA target prediction, and poorly conserved miRNAs lead to less reliable results. Using targetscan v.7.2, miR‐17‐3p, miR‐92a‐1‐5p, and miR‐181a‐1‐3p were considered part of the poorly conserved miRNA families, while the remaining miRNAs were part of the broadly conserved miRNA families. To strengthen the reliability of the results, the predicted targets from each of the tools were compared and only those identified by both were considered. Table 2 summarizes the number of predicted targets by each tool and the common ones, and Tables [Link], [Link], [Link], [Link], [Link], [Link], [Link], [Link] indicate the results of the bioinformatical analysis for each miRNA.

**Table 2:** Number of miRNA targets revealed by the bioinformatical target prediction, using targetscan v.7.2, diana tools microt‐cds v.5.0, or both.

miRNA	targetscan	diana tools microt‐cds	Common targets
miR‐194‐5p	305	551	169
miR‐802‐5p	251	666	140
miR‐17‐3p	4335	464	261
miR‐145a‐5p	539	706	224
miR‐338‐3p	342	745	158
miR‐205‐5p	374	925	207
miR‐92a‐1‐5p	1982	227	76
miR‐181a‐1‐3p	479	16	1

It is evident by the results presented in Table 2 that this type of bioinformatical prediction of the genes repressed by specific miRNAs generates huge amounts of information. Therefore, it would be very time‐ and cost‐consuming, and thus not reasonable, to experimentally validate all the targets. So, we performed a bibliographical search for the predicted targets. This search was directed to the proteins that have been related to BCBM, as well as with other processes that can be relevant for metastatic progression, including angiogenesis, disruption of the BBB, and invasion, among others. Besides proteins that have been described in BC, other types of cancers like brain tumors were also considered. Since miRNAs mainly work by negatively regulating gene expression, for the downregulated miRNAs (miR‐194‐5p, miR‐802‐5p, miR‐17‐3p, miR‐145‐5p, and miR‐338‐3p), we looked for oncogenic proteins, while for the upregulated miRNAs (miR‐205‐5p, miR‐92a‐1‐5p, and miR‐181a‐1‐3p), we looked for tumor‐suppressive proteins with a potential role in BCBM. Based on this bibliographic search, we selected miR‐802‐5p and miR‐194‐5p as the miRNAs of higher interest to further study, since these miRNAs have been shown to have tumor‐suppressive roles in different types of cancer. While miR‐802‐5p was shown to decrease  the proliferation of BCCs, through the downregulation of FoxM1 (Yuan and Wang, 2015), miR‐194‐5p has also been described to inhibit the proliferation and migration of BC, both *in vivo* and *in vitro*, though no specific targets were proposed (Le *et al.*, 2012). Therefore, the downregulation of miR‐802‐5p and miR‐194‐5p observed in our study at 3 days postinjection of 4T1 cells (Fig. 3B) is in line with a tumor suppressor role of these miRNAs. Based on the scores previously obtained, some targets with oncogenic functions were identified (Table 3). Among them was MEF2C, which appeared as a common target of both miR‐802‐5p and miR‐194‐5p, and was therefore selected for further studies.

**Table 3:** Predicted targets for microRNA (miR)‐802‐5p and miR‐194‐5p based on the bioinformatical analysis and a bibliographic research.

miRNA	Target gene	Name	Weighted context score	Aggregate PCT	miTG
miR‐802‐5p	*MSI1*	Musashi RNA‐binding protein‐1	−0.43	0.67	0.8566
	*RHOA*	RAS homolog family member A	−0.38	0.34	0.8175
	*TCF4*	Transcription factor 4	−0.18	0.19	0.9751
	*CDH11*	cadherin 11, type 2, OB‐cadherin (osteoblast)	−0.3	0.55	0.8580
	*CCND2*	Cyclin D2	−0.10	0.32	0.8698
	*MEF2C*	Myocyte enhancer factor 2C	−0.03	0.71	0.9523
miR‐194‐5p	*STAT1*	Signal transducer and activator of transcription 1	−0.45	< 0.10	0.7627
	*RAP2B*	RAP2B, member of RAS oncogene family	−0.44	0.79	0.8139
	*HBEGF*	Heparin‐binding EGF‐like growth factor	−0.25	0.69	0.993
	*AKT2*	v‐akt murine thymoma viral oncogene homolog 2	−0.15	0.53	0.7733
	*MEF2C*	Myocyte enhancer factor 2C	−0.16	0.55	0.7413
	*CHD2*	Cadherin 2, type 1, N‐cadherin (neuronal)	−0.17	0.55	0.7967

### 3.5 Validation of miR‐802‐5p and miR‐194‐5p by qPCR

**Figure 4:**
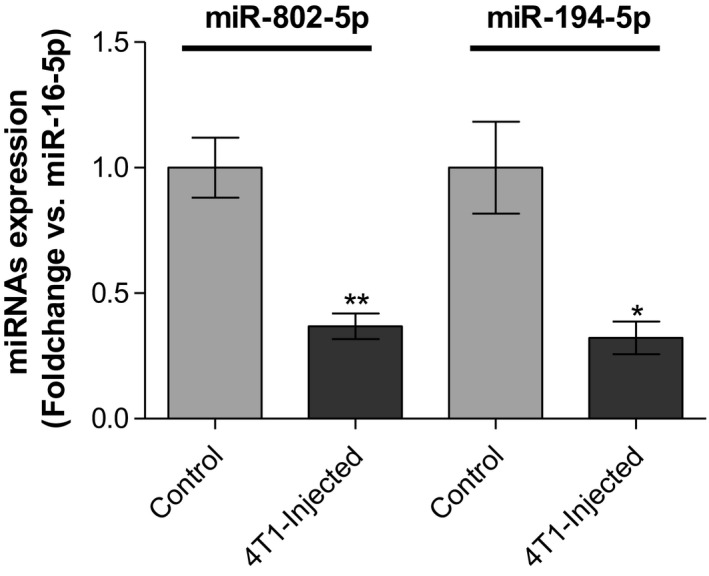
Validation of the next‐generation sequencing (NGS) results by quantitative real‐time PCR (qPCR) for microRNA (miR)‐802‐5p and miR‐194‐5p. Plasma samples were collected 3 days after injection of 4T1 cells or vehicle (control) in female Balb/c mice. Expression of miR‐802‐5p (*n* = 5) and miR‐194‐5p (*n* = 3) was evaluated by qPCR. Results are shown as mean values ± SD and expressed as fold change vs. miR‐16‐5p (endogenous control). *P < 0.05 and **P < 0.01 vs. control, by a two‐tailed unpaired Student’s *t*‐test.

NGS analysis is a very useful tool that enabled us to identify the changes in the plasma miRNome during brain metastasization. However, NGS of miRNAs is subject to sequencing errors and the search and removal of adapter sequences can also influence the results, with risk of false positives or negatives (Git *et al.*, 2010). Thus, to validate the aberrant expression of the selected miRNAs in plasma at 3 days, qPCR analysis of miR‐802‐5p and miR‐194‐5p was performed. Without a consensus about the ideal reference gene to study miRNA expression in plasma, we opted for miR‐16‐5p because it has been demonstrated to be stably expressed in plasma of several different mouse strains at different ages and disease conditions (Mi *et al.*, 2012). Moreover, Rinnerthaler *et al.* (2016) have demonstrated that miR‐16‐5p is also stably expressed in breast cancer tissues, both from primary and metastatic sites, indicating that it is a good housekeeping gene for our study, which was further confirmed by qPCR data. qPCR results (Fig. 4) allowed to validate the downregulation of miR‐802‐5p (*P* < 0.01) and of miR‐194‐5p (*P* < 0.05) in plasma of 4T1‐injected mice, when comparing to controls, at 3 days postinjection. Moreover, the qPCR analysis gave alterations in the same order of magnitude of NGS (Table 4), thus validating the downregulation of miR‐802‐5p and miR‐194‐5p. So, the results of both qPCR and NGS techniques revealed the downregulation of miR‐802‐5p and miR‐194‐5p, pointing to these miRNAs as efficient predictors of the upcoming occurrence of brain metastasis in cases of BC.

**Table 4:** Comparison of next‐generation sequencing (NGS) and quantitative real‐time polymerase chain reaction (qPCR) data regarding microRNAs (miRNAs or miR) aberrant expression in 4T1‐injected mice.

miRNA	NGS	qPCR	
	Fold change	Fold change	*P*‐value
miR‐802‐5p	0.1890	0.3682	< 0.01
miR‐194‐5p	0.4471	0.3180	< 0.05

### 3.6 MEF2C, a predicted target of both miR‐802‐5p and miR‐194‐5p, is highly expressed in breast cancer brain metastasis

MEF2C, a member of the family of transcription factors MEF2, was initially described to be activated during embryogenesis to regulate tissue‐specific gene expression and promote organ development, and is nowadays recognized to be also expressed during adult life in many types of cells, including neuronal and endothelial cells (Dong *et al.*, 2017). Although there is yet no proof of MEF2C involvement in the brain metastasization process, it has been proposed as a novel candidate oncogene, though evidence is limited to very few types of malignancies (Bai *et al.*, 2015; Zhang *et al.*, 2014).

**Figure 5:**
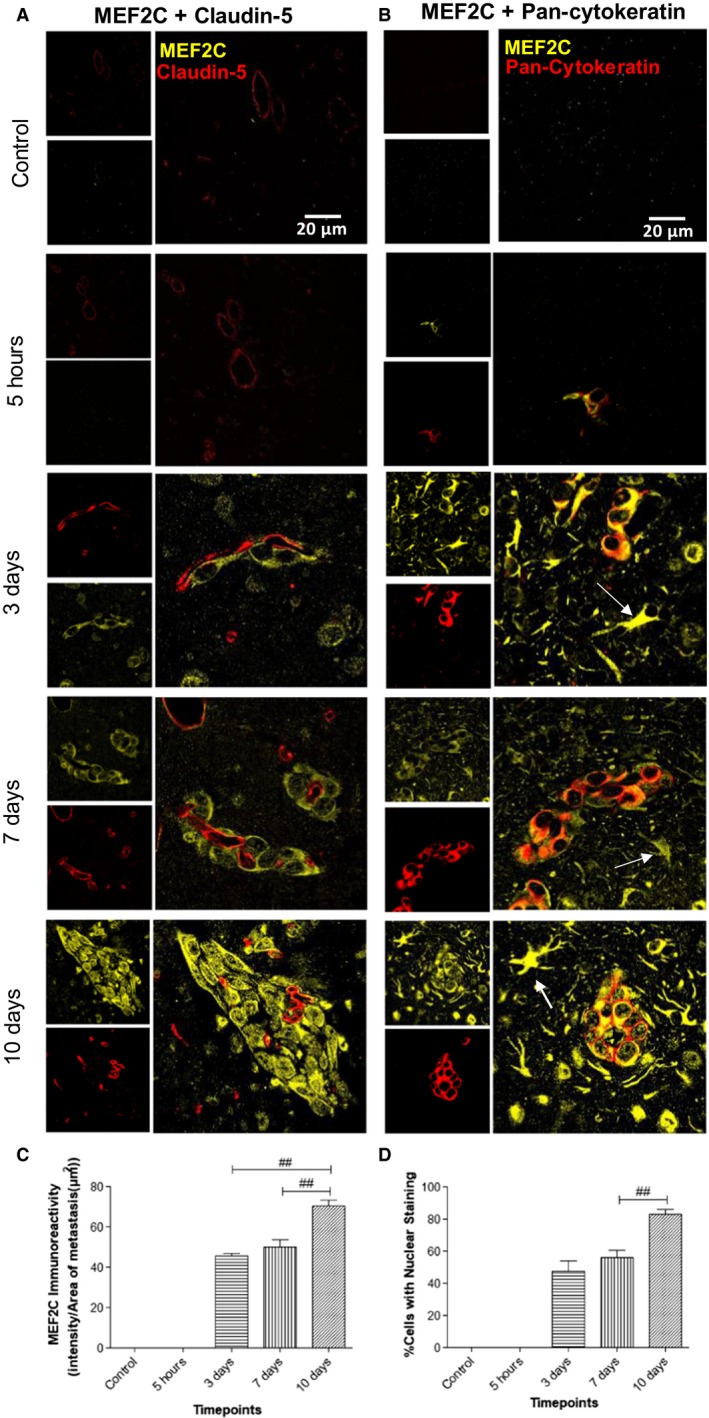
Myocyte enhancer factor 2C (MEF2C) expression in the brain parenchyma along brain metastasization. Immunofluorescence analysis of MEF2C in brain sections for different timepoints after 4T1 or vehicle (control) injection. Double labeling with claudin‐5 showed that MEF2C is not expressed in endothelial cells but only in perivascular cells (A). Double labeling immunofluorescence analysis of MEF2C and pan‐cytokeratin revealed that MEF2C‐positive cells are tumor cells (B). MEF2C labeling was also observed in other star‐shaped cells (arrows) (B). Semiquantitative analysis of MEF2C expression along time showed an increasing expression in tumor cells (C) and an increasing nuclear translocation of MEF2C (D). Results are expressed as mean ± SEM. A one‐way ANOVA, followed by the Bonferroni *post hoc* test, was used to evaluate the significant changes in the parameters between the different timepoints. ^##^ *P* < 0.01 between indicated groups.

The *MEF2C* gene was predicted as a target for miR‐802‐5p (Table 3). Although for the repression of *MEF2C* by miR‐802‐5p, the cumulative weighted context score ++ was low (cumulative weighted context score ++ = −0.03), the miTG was quite high (miTG = 0.9523), and, among the predicted targets, it was the one with the highest PCT (PCT = 0.71). Moreover, *MEF2C* was also a predicted target for miR‐194‐5p, the other miRNA that was validated as being downregulated in plasma (cumulative weighted context score ++ = −0.16; miTG = 0.74; PCT = 0.55). Altogether, this strengthens the possibility that MEF2C is involved in the brain metastasization process. In order to confirm this hypothesis, we assessed MEF2C expression in the brain parenchyma, during metastatic development (Fig. 5). Due to its previously described role in endothelial cells and angiogenesis (Maiti *et al.*, 2008), we studied its expression by immunofluorescence together with claudin‐5, a protein highly expressed in brain endothelial tight junctions (Cardoso *et al.*, 2010). Curiously, no colocalization was detected between claudin‐5 and MEF2C but MEF2C expression was found in perivascular cells at an early timepoint of metastatic development (3 days) (Fig. 5A). To confirm the nature of these cells, we double‐labeled MEF2C with pan‐cytokeratin, an epithelial marker expressed by malignant cells in the currently used model. Indeed, we observed a colocalization of MEF2C with pan‐cytokeratin and could confirm that from 3 days onwards, MEF2C is highly expressed in isolated malignant cells and in well‐established metastases (Fig. 5B). Analysis of MEF2C immunoreactivity showed a 24% increase in MEF2C expression/tumor area in metastasis throughout time, between 3 and 10 days (*P* < 0.01) and a 20% elevation between 7 and 10 days (*P* < 0.01) (Fig. 5C). These results showed that MEF2C is not expressed in endothelial cells, but it is highly expressed in brain metastatic cells and in well‐established metastasis. Moreover, MEF2C was increasingly expressed by tumor cells throughout time, which supports the involvement of this transcription factor in metastasis development.

### 3.7 MEF2C translocates to the nucleus in advanced stages of metastasis development

MEF2C is known to be a transcription factor, synthetized in the cytoplasm and translocated into the nucleus after activation (Liu *et al.*, 2018). Indeed, MEF2C was mostly concentrated in the cytoplasm at 3 and 7 days, while at 10 days following inoculation of malignant cells, it was homogenously expressed between the nucleus and the cytoplasm (Fig. 5). An analysis of the number of cells with nuclear staining per metastasis for the different timepoints showed that there was a significant increase of the percentage of cells with nuclear staining of approximately 29% between 3 and 10 days (*P* < 0.005) and of 32% between 7 and 10 days (*P* < 0.01) with no significant differences between 3 and 7 days (Fig. 5D). These results showed that there is a nuclear translocation of MEF2C with metastasis enlargement, indicating that MEF2C is more active in later stages of the metastatic development and pointing to this transcription factor as a new player in BCBM and a potential target for modulation.

### 3.8 MEF2C is highly expressed by peritumoral astrocytes

**Figure 6:**
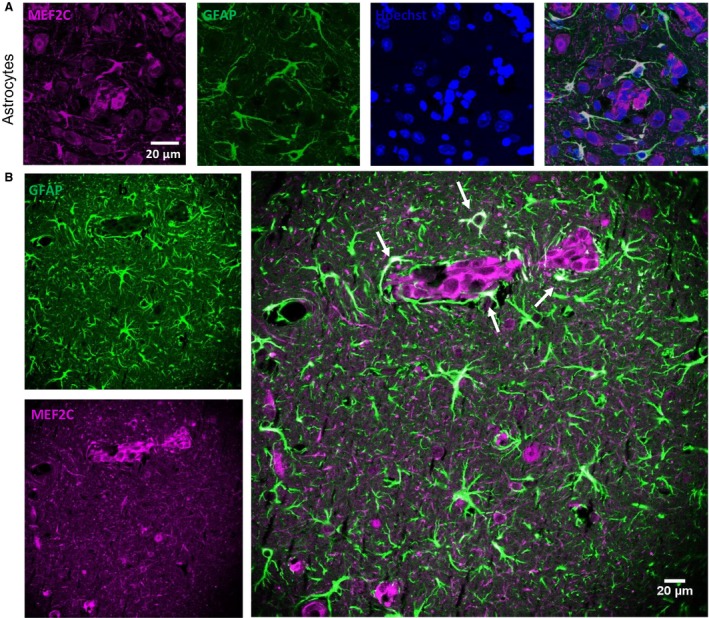
Myocyte enhancer factor 2C (MEF2C) expression in the peritumoral astrocytes. Brain sections from 4T1‐injected mice were analyzed, to study the expression of MEF2C in nontumoral cells, in close proximity to tumor cells. A double labeling for MEF2C and a marker for astrocytes (GFAP) was performed. Colocalization between MEF2C and GFAP was observed (A). Astrocytes that are close to the tumor (arrows) express MEF2C, while those that are further from the tumor only express GFAP (B). The different channels of the labeling are presented, as well as the merged pictures. Hoechst is labeling the nucleus.

Cells belonging to the neurovascular unit, including microglia and astrocytes, are known to interact with malignant cells after the transmigration through the BBB (Hasko *et al.*, 2019). An interesting observation from this study was the MEF2C expression in star‐shaped cells close to the metastatic lesions, as early as at 3 days, but not in controls (Fig. 5B). A double staining with GFAP, a marker for astrocytes, showed colocalization with MEF2C in such star‐shaped cells (Fig. 6A,B). Curiously, not all GFAP‐expressing cells expressed MEF2C, as MEF2C labeling was mainly found in GFAP‐expressing cells close to metastases (Fig. 6B), contrary to the ones that were further away from the tumors and that only expressed GFAP. These results reveal that peritumoral astrocytes, rather than distant ones, express MEF2C, pointing to a role of MEF2C in the crosstalk between tumor cells and astrocytes.

### 3.9 MEF2C is highly expressed in resected brain metastases from triple‐negative breast cancer patients

**Figure 7:**
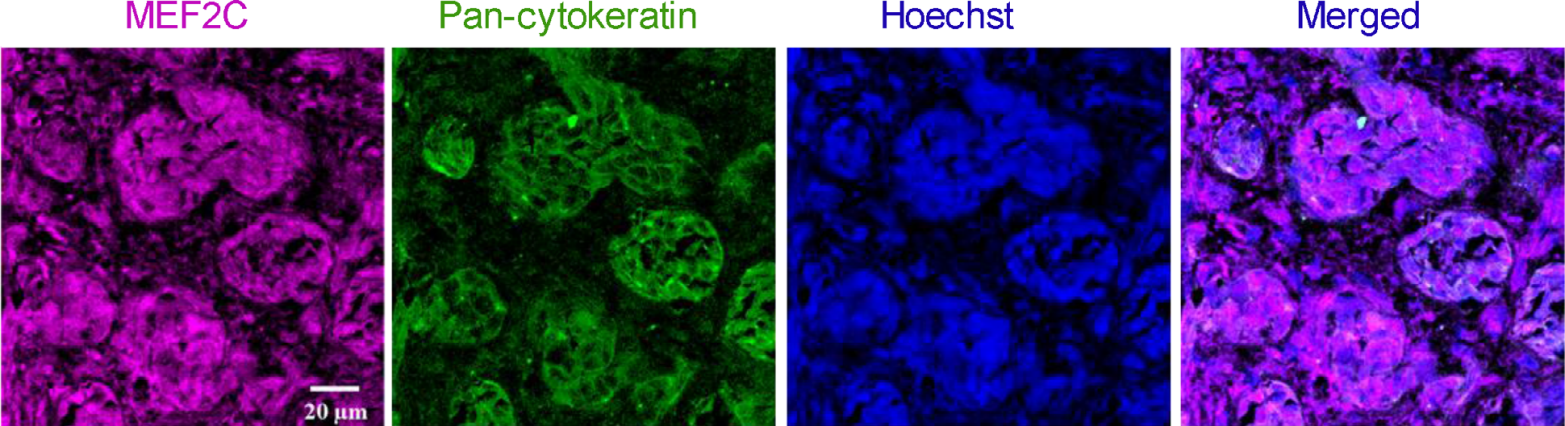
Myocyte enhancer factor 2C (MEF2C) expression in human brain metastases from triple‐negative breast carcinoma. Double labeling immunofluorescence analysis was performed for MEF2C with pan‐cytokeratin in sections from resected brain metastasis, from triple‐negative breast cancer patients. MEF2C shows colocalization with pan‐cytokeratin. The different channels of the labeling are presented, as well as the merged pictures. Hoechst is labeling the nucleus. Representative photographs from four different cases analyzed are shown.

To understand whether the results obtained in mouse brain sections are translatable to humans, we performed immunofluorescence analysis of sections from resected brain metastases, derived from triple‐negative BC in human patients. To distinguish tumoral from peripheral tissue, a double staining with pan‐cytokeratin was performed. As shown in Fig. 7, MEF2C expression was observed in metastasis, as corroborated by the double labeling with pan‐cytokeratin showing the colocalization of the transcription factor and the epithelial marker expressed by BC cells in brain metastasis. Altogether, these results show that MEF2C is expressed in triple‐negative breast carcinoma brain metastases.

## 4 Discussion

Due to the improved techniques for early detection of BC, together with the development of better therapeutic approaches, metastases are presently the major problem in oncology. Brain metastases are particularly challenging since they are usually detected upon appearance of clinical manifestations, which corresponds to a stage of the disease with poor prognosis, inasmuch as the BBB restricts the therapeutic options. Therefore, discovery of early biomarkers of brain metastasization and novel targets for modulation are needed for a precocious intervention and improvement of the patients’ outcome. Based on the fact that circulating miRNAs have increasingly been recognized as specific and sensitive biomarkers of different types of cancer (Wang *et al.*, 2018), we hypothesized that miRNAs are aberrantly expressed in plasma samples prior to the establishment of brain metastases and could potentially work as early biomarkers for BCBM. Our study revealed the early alteration in the expression levels of several miRNAs, and particularly the downregulation of miR‐802‐5p and miR‐194‐5p in plasma, and that their common target, MEF2C, is increasingly expressed in the brain along BC metastasis development. These findings pave the way for considering the altered miRNAs as potential biomarkers and MEF2C as a possible target for modulation to prevent or abrogate BCBM.

Among the different types of BC, triple‐negative BC is the most aggressive and has a high predisposition to develop brain metastases (Niwinska *et al.*, 2010). So, we used the murine mammary carcinoma 4T1 cell line, which is an aggressive triple‐negative tumor model that is highly tumorigenic and invasive, and metastasizes with a pattern very similar to that of human mammary cancer (Pulaski and Ostrand‐Rosenberg, 2001). The cells were injected in the common carotid artery, as an experimental model of brain metastasis (Zhang *et al.*, 2017), already applied successfully by us (Hasko *et al.*, 2019; Herman *et al.*, 2019). To ascertain brain specificity, metastatic progression was followed in different brain regions and peripheral organs. Whereas no metastases were observed in kidney or liver, they were detected in the lung. Importantly, the tumor area in the lung was similar to that of cerebellum, which was the least affected of the studied brain regions. These results indicate that in the experimental conditions used, BCCs are more prone to develop metastasis in the brain, particularly in the hippocampus, than in peripheral organs, rendering the used mouse model a suitable one to study brain metastasis from BC and its relationship with the miRNA alterations in peripheral circulation.

Analysis of the plasma miRNome by NGS confirmed our hypothesis that cell‐free miRNAs are deregulated during brain metastasis development and provided insights about the cell‐free miRNA signature in peripheral circulation along metastatic progression. Based on the pattern of metastasis, it is conceivable that the observed alterations in the plasma miRNA levels are mainly due to metastasization to brain, though not excluding any contribution from peripheral organs like the lungs. Moreover, the increasing number of deregulated miRNAs along time suggests an association with metastatic development. However, the main contributors to such altered levels remain to be determined, namely whether they result from an altered tumor cells’ activity or from a reaction from surrounding cells in the affected organ, presumably the brain. In line with the later possibility, there are evidences that upon tumor cell injection, the brain premetastatic niche can modulate the levels of the miRNAs to create a tumor‐favorable environment (Liu and Cao, 2016). Another relevant observation ensuing from NGS results is that miRNA levels are dynamic and are changing with time, and the same miRNAs that are deregulated in earlier stages of the tumor are not the same that are deregulated in more advanced stages, except for a small number of miRNAs that are deregulated throughout the metastatic development. This is supported by a recent study, showing that miRNA expression profiles can efficiently distinguish and categorize BC patients into early and advanced stages (Yerukala Sathipati and Ho, 2018).

Search for miRNAs with an aberrant expression prior to metastasis detection revealed miR‐194‐5p, miR‐802‐5p, miR‐17‐3p, miR‐145‐5p, and miR‐338‐3p, which are downregulated, and miR‐205‐5p, miR‐92a‐1‐5p, and miR‐181a‐1‐3p, which are upregulated. Considering that miRNAs mainly act by negatively regulating gene expression, it is predictable that those with a decreased expression mainly act as tumor suppressors, by downregulating oncogenes, while the upregulated ones should mainly work as oncogenes, downregulating tumor suppressor genes (Zhang *et al.*, 2007). Although the presently identified miRNAs have never been described in the development of BCBM, other studies support their tumor‐suppressive/oncogenic roles in other BC or metastatic conditions (De Cola *et al.*, 2015; Donzelli *et al.*, 2015; Le *et al.*, 2012; Lin *et al.*, 2013; Taylor *et al.*, 2013; Ye *et al.*, 2014; Yuan and Wang, 2015). Among those miRNAs, miR‐802‐5p and miR‐194‐5p emerged as the most promising, by being downregulated prior to metastasis development, and potentially being related to the upregulation of oncogenic proteins, as revealed by bioinformatical target prediction and bibliographic search.

The downregulation of miR‐802‐5p has been described for different types of cancer. In prostate cancer, this miRNA inhibited epithelial–mesenchymal transition, an essential step for metastasis development, by downregulating *flotillin‐2*, a known downstream gene of p53. Accordingly, its forced expression led to a decrease in mesenchymal markers and suppressed metastatic ability of cancer cells (Wang *et al.*, 2017). MiR‐802‐5p downregulation was also shown to increase Wnt activation in pancreatic adenocarcinoma (Muller *et al.*, 2015). Regarding miR‐802‐5p in BC, it was shown to have a lower expression in BCCs comparing to normal breast epithelial cells, and that its overexpression decreased BC proliferation in both *in vitro* and *in vivo* experiments, through downregulation of *FoxM1* (Yuan and Wang, 2015). These evidences support that miR‐802‐5p can have a tumor‐suppressive action and that its downregulation may play a role in the metastatic progression.

Regarding miR‐194‐5p, ambiguous results are found in the literature. In fact, there are reports of elevated levels in esophageal squamous cell carcinoma (Wu *et al.*, 2014) and in prostate cancer patients who subsequently experienced recurrence (Selth *et al.*, 2013). In contrast, another study showed significantly lower miR‐194‐5p in colorectal cancer patients than in control subjects, and an inverse correlation with the advanced stages, suggesting that reduced levels of the miRNA could serve as diagnostic and prognostic biomarkers for patients with colorectal cancer (Basati *et al.*, 2016). In BC, miR‐194‐5p was described as having tumor‐suppressive roles by inhibiting proliferation and migration *in vitro* and *in vivo* (Le *et al.*, 2012). However, a recent paper demonstrated that miR‐194‐5p enhances cell proliferation, migration, and invasion in different BC cell lines via Wnt/β‐catenin pathway regulation (Yang *et al.*, 2018). These contradicting reports may be a consequence of the multitude of genes and functions that one single miRNA can regulate and of the multiple factors and cellular contexts that influence their expression (Dykxhoorn, 2010). However, none of these studies analyzed plasma levels of miR‐194‐5p prior to development of brain metastasis. Thus, they do not argue against the herewith presented downregulation of miR‐194‐5p, which in turn is in line with its predicted targets that have been described as oncogenes in BC or other types of cancer and to promote metastatic development.

The dysregulation of a miRNA also implies the dysregulation of its targets. Thus, having found two miRNAs with an altered expression, the bioinformatical analysis was used as a way to have better insights about the mechanisms and pathways in which these miRNAs can be involved during brain metastasis development. Among these targets emerged *MEF2*C, related to both miR‐802‐5p and miR‐194‐5p. *MEF2C* has recently been proposed as a new oncogene, promoting metastasis in pancreatic adenocarcinoma by inducing MMP10 transcription (Zhang *et al.*, 2014) and mediating VEGF induction of vasculogenic mimicry, migration, and invasion in hepatocellular carcinoma (Bai *et al.*, 2015). MEF2C is a member of the MEF2 protein family that was initially associated with the development of heart and muscle and is now known to have close connections with biological features that are characteristic of cancer, like uncontrolled proliferation and enhancement of invasion (Chen *et al.*, 2017). Accordingly, one of its members, MEF2D, has been related to lung cancer (Zhang *et al.*, 2015), hepatocellular carcinoma (Ma *et al.*, 2014), and glioma (Zhao *et al.*, 2015), with its expression regulated by miR‐1244, miR‐122, and miR‐18a, respectively. Besides miRNAs, signaling pathways such as Ca^2+^ signaling pathway, EGFR, MAP kinase, Wnt, and PI3K/Akt signaling pathways can activate MEF2 (Chen *et al.*, 2017). Moreover, a molecular regulatory loop whereby MEF2D regulates miR‐1244 was observed in lung cancer (Zhang *et al.*, 2015). As far as ME2C is concerned, it demonstrated its activation by the MAPK p38 in inflammation in monocytic cells (Han *et al.*, 1997), whereas downregulation of the MUC4/ErbB2/p38/MEF2C‐dependent pathway was shown to suppress invasion and metastasis of pancreatic ductal adenocarcinoma (Zhang *et al.*, 2014). MEF2C expression was also shown in BC, with studies showing its upregulation in MCF7 BCCs occurring together with upregulation of apoptosis‐related cysteine peptidase gene, one of the initiator caspases (Motaghed *et al.*, 2014). MEF2C expression was also reported in primary BC tissues and shown to be activated by p38MAPK in metastatic BC (Ostrander *et al.*, 2007). Our analysis of the brain parenchyma revealed that MEF2C is highly expressed in BCBM and that its expression increases with tumor progression. This had never been described before but suggests an oncogenic role for MEF2C in promoting BCBM. Furthermore, a nuclear translocation of MEF2C was observed in later stages of tumor progression. Since MEF2C is a transcription factor, the natural assumption is that this translocation means a higher activation of MEF2C to promote its target genes transcription, like the aforementioned MMP10 and VEGF, and support metastatic growth. Interestingly, a nuclear translocation of MEF2C was also observed in hepatocellular carcinoma, in which the cytosolic location was associated with the proliferation biomarker Ki‐67, while the nuclear location was correlated with the angiogenesis‐associated biomarker CD31, suggesting that the subcellular distribution dictates the overall effect of MEF2C (Bai *et al.*, 2015). The proliferative activity here detected by Ki‐67, together with the MEF2C inhibition of apoptosis observed in endothelial cells (Hayashi *et al.*, 2004) and developing neurons (Okamoto *et al.*, 2000), may further contribute to metastasis development. These observations provide a basis for further studying the contribution of MEF2C to the brain metastatic process and the underlying signaling pathways. It would also be interesting to analyze whether MEF2C is phosphorylated as reported in acute myeloid leukemia where phosphorylation appeared to induce apoptosis resistance and its inhibition reverted chemotherapy resistance (Brown *et al.*, 2018). Moreover, by revealing MEF2C in BCBM, they point to the relevance of further studying this transcription factor both as a prognostic biomarker and as a potential target for modulation.

Upregulation of MEF2C found in mouse samples was validated by the observation of a marked expression of the protein in resected brain metastasis from BC patients, which increases the relevance of the present study. Interestingly, overexpression of *MEF2C* was also observed in pretreatment bone marrow specimens from acute myeloid leukemia patients, which was correlated with MEF2C protein expression, suggesting a contribution to the aggressive nature of at least some subtypes of the disease (Laszlo *et al.*, 2015). Moreover, *MEF2C* was considered one of the driving oncogenes in acute lymphoblastic leukemia and demonstrated to be overexpressed in T cells from disease patients (Colomer‐Lahiguera *et al.*, 2017). In the same study, *MEF2C* dysregulation was correlated with a poor response of the patients to glucocorticoid therapy. Based on these observations, it is conceivable that the expression of MEF2C in human brain metastasis from BC patients contributes to the poor prognosis by compromising the response to the therapy.

Astrocytes are key components of the brain metastatic microenvironment and are determinants of malignant cell fate in the brain. Although, initially, astrocytes have an harmful role for tumor cells, in later stages astrocytes share a bidirectional communication with tumor cells and produce proteases, growth factors, and inflammatory cytokines, to support tumor growth (Wilhelm *et al.*, 2019). MEF2C expression has previously been described as a regulator of the inflammatory response by microglia, during aging (Deczkowska *et al.*, 2017), but its expression in astrocytes has not been described, yet. Here, we show that astrocytes start expressing MEF2C after tumor cell extravasation. Interestingly, mainly peritumoral astrocytes express MEF2C, contrarily to non‐peritumoral astrocytes. This can indicate that MEF2C is involved in the crosstalk between astroglial cells and tumor cells during BCBM formation, to support tumor growth, which deserves further studies.

## 5 Conclusions

With this work, we showed that during BCBM formation, circulating miRNAs are deregulated in a time‐dependent manner, with some of them deregulated even before brain metastasis detection. Prospective studies in patients will clarify whether decrease in miR‐802 and miR‐194 predicts the appearance of secondary tumors in the central nervous system in humans. Our study might thus provide a basis for the development of novel diagnostic strategies based on the detection of plasma miRNAs as biomarkers of brain metastases.

We also described for the first time the overexpression of MEF2C and its translocation into the nucleus, which suggest a supporting role in the development of brain metastasis from BC for this transcription factor, predicted to be a target of both miR‐802‐5p and miR‐194‐5p. Noteworthy, its overexpression in peritumoral astrocytes may underline the interplay between these glial cells and the malignant ones. Finally, the unequivocal expression of MEF2C in resected brain metastasis from BC patients allows the translation of the findings obtained in a mouse model to humans.

## Conflict of interest

The authors declare no conflict of interest.

## Author contributions

MAB conceived the study and gathered together the team. MS, JH, KM, and SJM performed the experiments. MAB, SAB, IW, and IAK contributed to experimental development control and validation, as well as to data analysis. RM, MV, ZR, and LT provided resources for study development and data acquisition. MS prepared the figures and the original draft. All authors provided critical feedback and reviewed and edited the manuscript. MAB, IW, and IAK obtained the funding for the study accomplishment.

## Supporting information


**Table S1.** MiRNAs found to be deregulated at 7 days, by NGS analysis

**Table S2.** MiRNAs found to be deregulated at 10 days, by NGS analysis.

**Table S3.** Results of the target prediction for miR‐194‐5p using Target Scan v.7.2 and Diana Tool MicroT‐CDS v.5.0.

**Table S4.** Results of the target prediction for miR‐802‐5p using TargetScan v.7.2 and diana tools MicroT‐CDS v.5.0.

**Table S5.** Results of the target prediction for miR‐17‐3p using TargetScan v.7.2 and diana tools MicroT‐CDS v.5.0.

**Table S6.** Results of the target prediction for miR‐145‐5p using TargetScan v.7.2. and diana tools MicroT‐CDS v.5.0.

**Table S7.** Results of the target prediction for miR‐338‐3p using TargetScan v.7.2 and diana tools MicroT‐CDS v.5.0.

**Table S8.** Results of the target prediction for miR‐205‐5p using TargetScan v.7.2. and diana tools MicroT‐CDS v.5.0.

**Table S9.** Results of the target prediction for miR‐92a‐1‐5p using TargetScan v.7.2 and Diana MicroT‐CDS v.5.0.

**Table S10.** Results of the target prediction for miR‐181a‐3p using TargetScan v.7.2. and diana tools MicroT‐CDS v.5.0.

## References

[mol212632-bib-0001] Agarwal V , Bell GW , Nam JW and Bartel DP (2015) Predicting effective microRNA target sites in mammalian mRNAs. eLife 4, 1–38. 10.7554/eLife.05005 PMC4532895 26267216

[mol212632-bib-0002] Bai XL , Zhang Q , Ye LY , Liang F , Sun X , Chen Y , Hu QD , Fu QH , Su W , Chen Z *et al* (2015) Myocyte enhancer factor 2C regulation of hepatocellular carcinoma via vascular endothelial growth factor and Wnt/β‐catenin signaling. Oncogene 34, 4089–4097. 25328135 10.1038/onc.2014.337

[mol212632-bib-0003] Basati G , Razavi AE , Pakzad I and Malayeri FA (2016) Circulating levels of the miRNAs, miR‐194, and miR‐29b, as clinically useful biomarkers for colorectal cancer. Tumour Biol 37, 1781–1788. 26318304 10.1007/s13277-015-3967-0

[mol212632-bib-0004] Brown FC , Still E , Koche RP , Yim CY , Takao S , Cifani P , Reed C , Gunasekera S , Ficarro SB , Romanienko P *et al* (2018) MEF2C phosphorylation is required for chemotherapy resistance in acute myeloid leukemia. Cancer Discov 8, 478–497. 29431698 10.1158/2159-8290.CD-17-1271 PMC5882571

[mol212632-bib-0005] Cardoso FL , Brites D and Brito MA (2010) Looking at the blood‐brain barrier: molecular anatomy and possible investigation approaches. Brain Res Rev 64, 328–363. 20685221 10.1016/j.brainresrev.2010.05.003

[mol212632-bib-0006] Chen X , Ba Y , Ma L , Cai X , Yin Y , Wang K , Guo J , Zhang Y , Chen J , Guo X *et al* (2008) Characterization of microRNAs in serum: a novel class of biomarkers for diagnosis of cancer and other diseases. Cell Res 18, 997–1006. 18766170 10.1038/cr.2008.282

[mol212632-bib-0007] Chen X , Gao B , Ponnusamy M , Lin Z and Liu J (2017) MEF2 signaling and human diseases. Oncotarget 8, 112152–112165. 29340119 10.18632/oncotarget.22899 PMC5762387

[mol212632-bib-0008] Colomer‐Lahiguera S , Pisecker M , Konig M , Nebral K , Pickl WF , Kauer MO , Haas OA , Ullmann R , Attarbaschi A , Dworzak MN *et al* (2017) MEF2C‐dysregulated pediatric T‐cell acute lymphoblastic leukemia is associated with CDKN1B deletions and a poor response to glucocorticoid therapy. Leuk Lymphoma 58, 2895–2904. 28482719 10.1080/10428194.2017.1312383

[mol212632-bib-0009] De Cola A , Volpe S , Budani MC , Ferracin M , Lattanzio R , Turdo A , D'Agostino D , Capone E , Stassi G , Todaro M *et al* (2015) miR‐205‐5p‐mediated downregulation of ErbB/HER receptors in breast cancer stem cells results in targeted therapy resistance. Cell Death Dis 6, e1823–e1823. 26181203 10.1038/cddis.2015.192 PMC4650737

[mol212632-bib-0010] Deczkowska A , Matcovitch‐Natan O , Tsitsou‐Kampeli A , Ben‐Hamo S , Dvir‐Szternfeld R , Spinrad A , Singer O , David E , Winter DR , Smith LK *et al* (2017) Mef2C restrains microglial inflammatory response and is lost in brain ageing in an IFN‐I‐dependent manner. Nat Commun 8, 717. 28959042 10.1038/s41467-017-00769-0 PMC5620041

[mol212632-bib-0011] Dong C , Yang XZ , Zhang CY , Liu YY , Zhou RB , Cheng QD , Yan EK and Yin DC (2017) Myocyte enhancer factor 2C and its directly‐interacting proteins: a review. Prog Biophys Mol Biol 126, 22–30. 28163053 10.1016/j.pbiomolbio.2017.02.002

[mol212632-bib-0012] Donzelli S , Mori F , Bellissimo T , Sacconi A , Casini B , Frixa T , Roscilli G , Aurisicchio L , Facciolo F , Pompili A *et al* (2015) Epigenetic silencing of miR‐145‐5p contributes to brain metastasis. Oncotarget 6, 35183–35201. 26440147 10.18632/oncotarget.5930 PMC4742098

[mol212632-bib-0013] Dykxhoorn DM (2010) MicroRNAs and metastasis: little RNAs go a long way. Cancer Res 70, 6401–6406. 20663901 10.1158/0008-5472.CAN-10-1346 PMC2922433

[mol212632-bib-0014] Ferlay J , Soerjomataram I , Dikshit R , Eser S , Mathers C , Rebelo M , Parkin DM , Forman D and Bray F (2015) Cancer incidence and mortality worldwide: sources, methods and major patterns in GLOBOCAN 2012. Int J Cancer 136, E359–E386. 25220842 10.1002/ijc.29210

[mol212632-bib-0015] Friedman RC , Farh KK , Burge CB and Bartel DP (2009) Most mammalian mRNAs are conserved targets of microRNAs. Genome Res 19, 92–105. 18955434 10.1101/gr.082701.108 PMC2612969

[mol212632-bib-0016] Git A , Dvinge H , Salmon‐Divon M , Osborne M , Kutter C , Hadfield J , Bertone P and Caldas C (2010) Systematic comparison of microarray profiling, real‐time PCR, and next‐generation sequencing technologies for measuring differential microRNA expression. RNA 16, 991–1006. 20360395 10.1261/rna.1947110 PMC2856892

[mol212632-bib-0017] Han J , Jiang Y , Li Z , Kravchenko VV and Ulevitch RJ (1997) Activation of the transcription factor MEF2C by the MAP kinase p38 in inflammation. Nature 386, 296–299. 9069290 10.1038/386296a0

[mol212632-bib-0018] Hasko J , Fazakas C , Molnar K , Meszaros A , Patai R , Szabo G , Erdelyi F , Nyul‐Toth A , Gyori F , Kozma M *et al* (2019) Response of the neurovascular unit to brain metastatic breast cancer cells. Acta Neuropathol Commun 7, 133. 31426859 10.1186/s40478-019-0788-1 PMC6699134

[mol212632-bib-0019] Hayashi M , Kim SW , Imanaka‐Yoshida K , Yoshida T , Abel ED , Eliceiri B , Yang Y , Ulevitch RJ and Lee JD (2004) Targeted deletion of BMK1/ERK5 in adult mice perturbs vascular integrity and leads to endothelial failure. J Clin Invest 113, 1138–1148. 15085193 10.1172/JCI19890 PMC385403

[mol212632-bib-0020] He Y , Lin J , Kong D , Huang M , Xu C , Kim TK , Etheridge A , Luo Y , Ding Y and Wang K (2015) Current state of circulating microRNAs as cancer biomarkers. Clin Chem 61, 1138–1155. 26319452 10.1373/clinchem.2015.241190

[mol212632-bib-0021] Herman H , Fazakas C , Hasko J , Molnar K , Meszaros A , Nyul‐Toth A , Szabo G , Erdelyi F , Ardelean A , Hermenean A *et al* (2019) Paracellular and transcellular migration of metastatic cells through the cerebral endothelium. J Cell Mol Med 23, 2619–2631. 30712288 10.1111/jcmm.14156 PMC6433661

[mol212632-bib-0022] Karachaliou N , Mayo‐de‐Las‐Casas C , Molina‐Vila MA and Rosell R (2015) Real‐time liquid biopsies become a reality in cancer treatment. Ann Transl Med 3, 36. 25815297 10.3978/j.issn.2305-5839.2015.01.16 PMC4356857

[mol212632-bib-0023] Kotecki N , Lefranc F , Devriendt D and Awada A (2018) Therapy of breast cancer brain metastases: challenges, emerging treatments and perspectives. Ther Adv Med Oncol 10, 1–10. 10.1177/1758835918780312 PMC6024336 29977353

[mol212632-bib-0024] Laszlo GS , Alonzo TA , Gudgeon CJ , Harrington KH , Kentsis A , Gerbing RB , Wang Y‐C , Ries RE , Raimondi SC , Hirsch BA *et al* (2015) High expression of myocyte enhancer factor 2C (MEF2C) is associated with adverse‐risk features and poor outcome in pediatric acute myeloid leukemia: a report from the Children’s Oncology Group. J Hematol Oncol 8, 115–115. 26487643 10.1186/s13045-015-0215-4 PMC4618184

[mol212632-bib-0025] Le XF , Almeida MI , Mao W , Spizzo R , Rossi S , Nicoloso MS , Zhang S , Wu Y , Calin GA and Bast RC Jr (2012) Modulation of MicroRNA‐194 and cell migration by HER2‐targeting trastuzumab in breast cancer. PLoS ONE 7, e41170. 22829924 10.1371/journal.pone.0041170 PMC3400637

[mol212632-bib-0026] Leone JP , Lee AV and Brufsky AM (2015) Prognostic factors and survival of patients with brain metastasis from breast cancer who underwent craniotomy. Cancer Med 4, 989–994. 25756607 10.1002/cam4.439 PMC4529337

[mol212632-bib-0027] Lim LP , Lau NC , Garrett‐Engele P , Grimson A , Schelter JM , Castle J , Bartel DP , Linsley PS and Johnson JM (2005) Microarray analysis shows that some microRNAs downregulate large numbers of target mRNAs. Nature 433, 769–773. 15685193 10.1038/nature03315

[mol212632-bib-0028] Lin HY , Chiang CH and Hung WC (2013) STAT3 upregulates miR‐92a to inhibit RECK expression and to promote invasiveness of lung cancer cells. Br J Cancer 109, 731–738. 23820254 10.1038/bjc.2013.349 PMC3738132

[mol212632-bib-0029] Liu Y and Cao X (2016) Characteristics and Significance of the Pre‐metastatic Niche. Cancer Cell 30, 668–681. 27846389 10.1016/j.ccell.2016.09.011

[mol212632-bib-0030] Liu Y , Li P , Fan L and Wu M (2018) The nuclear transportation routes of membrane‐bound transcription factors. Cell Commun Signal 16, 12. 29615051 10.1186/s12964-018-0224-3 PMC5883603

[mol212632-bib-0031] Ma L , Liu J , Liu L , Duan G , Wang Q , Xu Y , Xia F , Shan J , Shen J , Yang Z *et al* (2014) Overexpression of the transcription factor MEF2D in hepatocellular carcinoma sustains malignant character by suppressing G2‐M transition genes. Cancer Res 74, 1452–1462. 24390737 10.1158/0008-5472.CAN-13-2171

[mol212632-bib-0032] Maiti D , Xu Z and Duh EJ (2008) Vascular endothelial growth factor induces MEF2C and MEF2‐dependent activity in endothelial cells. Invest Ophthalmol Vis Sci 49, 3640–3648. 18450586 10.1167/iovs.08-1760 PMC4519038

[mol212632-bib-0033] Mi QS , Weiland M , Qi RQ , Gao XH , Poisson LM and Zhou L (2012) Identification of mouse serum miRNA endogenous references by global gene expression profiles. PLoS ONE 7, e31278. 22348064 10.1371/journal.pone.0031278 PMC3277497

[mol212632-bib-0034] Motaghed M , Al‐Hassan FM and Hamid SS (2014) Thymoquinone regulates gene expression levels in the estrogen metabolic and interferon pathways in MCF7 breast cancer cells. Int J Mol Med 33, 8–16. 24270600 10.3892/ijmm.2013.1563 PMC3868490

[mol212632-bib-0035] Muller S , Raulefs S , Bruns P , Afonso‐Grunz F , Plotner A , Thermann R , Jager C , Schlitter AM , Kong B , Regel I *et al* (2015) Next‐generation sequencing reveals novel differentially regulated mRNAs, lncRNAs, miRNAs, sdRNAs and a piRNA in pancreatic cancer. Mol Cancer 14, 94. 25910082 10.1186/s12943-015-0358-5 PMC4417536

[mol212632-bib-0036] Niwinska A , Murawska M and Pogoda K (2010) Breast cancer brain metastases: differences in survival depending on biological subtype, RPA RTOG prognostic class and systemic treatment after whole‐brain radiotherapy (WBRT). Ann Oncol 21, 942–948. 19840953 10.1093/annonc/mdp407

[mol212632-bib-0037] Okamoto S , Krainc D , Sherman K and Lipton SA (2000) Antiapoptotic role of the p38 mitogen‐activated protein kinase‐myocyte enhancer factor 2 transcription factor pathway during neuronal differentiation. Proc Natl Acad Sci USA 97, 7561–7566. 10852968 10.1073/pnas.130502697 PMC16585

[mol212632-bib-0038] Ostrander JH , Daniel AR , Lofgren K , Kleer CG and Lange CA (2007) Breast tumor kinase (protein tyrosine kinase 6) regulates heregulin‐induced activation of ERK5 and p38 MAP kinases in breast cancer cells. Cancer Res 67, 4199–4209. 17483331 10.1158/0008-5472.CAN-06-3409

[mol212632-bib-0039] Paraskevopoulou MD , Georgakilas G , Kostoulas N , Vlachos IS , Vergoulis T , Reczko M , Filippidis C , Dalamagas T and Hatzigeorgiou A (2013) DIANA‐microT web server v5. 0: service integration into miRNA functional analysis workflows. Nucleic Acids Res 41(W1), W169–W173. 23680784 10.1093/nar/gkt393 PMC3692048

[mol212632-bib-0040] Pulaski BA and Ostrand‐Rosenberg S (2001) Mouse 4T1 breast tumor model. Curr Protoc Immunol 10.1002/0471142735.im2002s39 18432775

[mol212632-bib-0041] Riffo‐Campos ÁL , Riquelme I and Brebi‐Mieville P (2016) Tools for sequence‐based miRNA target prediction: What to choose? Int J Mol Sci 17, 1987. 10.3390/ijms17121987 PMC5187787 27941681

[mol212632-bib-0042] Rinnerthaler G , Hackl H , Gampenrieder SP , Hamacher F , Hufnagl C , Hauser‐Kronberger C , Zehentmayr F , Fastner G , Sedlmayer F , Mlineritsch B *et al* (2016) miR‐16‐5p Is a Stably‐expressed housekeeping MicroRNA in breast cancer tissues from primary tumors and from metastatic sites. Int J Mol Sci 17, 156. 10.3390/ijms17020156 PMC4783890 26821018

[mol212632-bib-0043] Rosenfeld N , Aharonov R , Meiri E , Rosenwald S , Spector Y , Zepeniuk M , Benjamin H , Shabes N , Tabak S , Levy A *et al* (2008) MicroRNAs accurately identify cancer tissue origin. Nat Biotechnol 26, 462–469. 18362881 10.1038/nbt1392

[mol212632-bib-0044] Selth LA , Townley SL , Bert AG , Stricker PD , Sutherland PD , Horvath LG , Goodall GJ , Butler LM and Tilley WD (2013) Circulating microRNAs predict biochemical recurrence in prostate cancer patients. Br J Cancer 109, 641–650. 23846169 10.1038/bjc.2013.369 PMC3738112

[mol212632-bib-0045] Taylor MA , Sossey‐Alaoui K , Thompson CL , Danielpour D and Schiemann WP (2013) TGF‐ß upregulates miR‐181a expression to promote breast cancer metastasis. J Clin Invest 123, 150–163. 23241956 10.1172/JCI64946 PMC3533297

[mol212632-bib-0046] Torre LA , Siegel RL , Ward EM and Jemal A (2016) Global cancer incidence and mortality rates and trends–an update. Cancer Epidemiol Biomark Prev 25, 16–27. 10.1158/1055-9965.EPI-15-0578 26667886

[mol212632-bib-0047] Wahid F , Shehzad A , Khan T and Kim YY (2010) MicroRNAs: Synthesis, mechanism, function, and recent clinical trials. Biochim Biophys Acta 1803, 1231–1243. 20619301 10.1016/j.bbamcr.2010.06.013

[mol212632-bib-0048] Wang D , Lu G , Shao Y and Xu D (2017) MicroRNA‐802 inhibits epithelial‐mesenchymal transition through targeting Flotillin‐2 in human prostate cancer. Biosci Rep 37, BSR20160521. 10.1042/BSR20160521 PMC5350603 28188157

[mol212632-bib-0049] Wang H , Peng R , Wang J , Qin Z and Xue L (2018) Circulating microRNAs as potential cancer biomarkers: the advantage and disadvantage. Clin Epigenetics 10, 59. 29713393 10.1186/s13148-018-0492-1 PMC5913875

[mol212632-bib-0050] Wilhelm I , Fazakas C , Molnár K , Végh AG , Haskó J and Krizbai IA (2019) Foe or friend? Janus‐faces of the neurovascular unit in the formation of brain metastases. J Cereb Blood Flow Metab 38, 563–587. 10.1177/0271678X17732025 PMC5888855 28920514

[mol212632-bib-0051] Winkler F (2015) The brain metastatic niche. J Mol Med 93, 1213–1220. 26489608 10.1007/s00109-015-1357-0

[mol212632-bib-0052] Wu C , Wang C , Guan X , Liu Y , Li D , Zhou X , Zhang Y , Chen X , Wang J , Zen K *et al* (2014) Diagnostic and prognostic implications of a serum miRNA panel in oesophageal squamous cell carcinoma. PLoS ONE 9, e92292. 24651474 10.1371/journal.pone.0092292 PMC3961321

[mol212632-bib-0053] Yang F , Xiao Z and Zhang S (2018) Knockdown of miR‐194‐5p inhibits cell proliferation, migration and invasion in breast cancer by regulating the Wnt/beta‐catenin signaling pathway. Int J Mol Med 42, 3355–3363. 30272253 10.3892/ijmm.2018.3897 PMC6202083

[mol212632-bib-0054] Ye F , Tang H , Liu Q , Xie X , Wu M , Liu X , Chen B and Xie X (2014) miR‐200b as a prognostic factor in breast cancer targets multiple members of RAB family. J Transl Med 12, 17. 24447584 10.1186/1479-5876-12-17 PMC3898994

[mol212632-bib-0055] Yerukala Sathipati S and Ho S‐Y (2018) Identifying a miRNA signature for predicting the stage of breast cancer. Sci Rep 8, 16138. 30382159 10.1038/s41598-018-34604-3 PMC6208346

[mol212632-bib-0056] Yuan F and Wang W (2015) MicroRNA‐802 suppresses breast cancer proliferation through downregulation of FoxM1. Mol Med Rep 12, 4647–4651. 26080894 10.3892/mmr.2015.3921

[mol212632-bib-0057] Zhang B , Pan X , Cobb GP and Anderson TA (2007) microRNAs as oncogenes and tumor suppressors. Dev Biol 302, 1–12. 16989803 10.1016/j.ydbio.2006.08.028

[mol212632-bib-0058] Zhang C , Lowery FJ and Yu D (2017) Intracarotid cancer cell injection to produce mouse models of brain metastasis. J Vis Exp 120. 10.3791/55085 PMC5409267 28287553

[mol212632-bib-0059] Zhang JJ , Zhu Y , Xie KL , Peng YP , Tao JQ , Tang J , Li Z , Xu ZK , Dai CC , Qian ZY *et al* (2014) Yin Yang‐1 suppresses invasion and metastasis of pancreatic ductal adenocarcinoma by downregulating MMP10 in a MUC4/ErbB2/p38/MEF2C‐dependent mechanism. Mol Cancer 13, 1–17. 24884523 10.1186/1476-4598-13-130 PMC4047260

[mol212632-bib-0060] Zhang R , Zhang Y and Li H (2015) miR‐1244/myocyte enhancer factor 2D regulatory loop contributes to the growth of lung carcinoma. DNA Cell Biol 34, 692–700. 26355845 10.1089/dna.2015.2915

[mol212632-bib-0061] Zhao YY , Zhao LN , Wang P , Miao YS , Liu YH , Wang ZH , Ma J , Li Z , Li ZQ and Xue YX (2015) Overexpression of miR‐18a negatively regulates myocyte enhancer factor 2D to increase the permeability of the blood‐tumor barrier via Kruppel‐like factor 4‐mediated downregulation of zonula occluden‐1, claudin‐5, and occludin. J Neurosci Res 93, 1891–1902. 26356851 10.1002/jnr.23628

