# Supplementary material for: Downregulation of circulating miR 802‐5p and miR 194‐5p and upregulation of brain MEF2C along breast cancer brain metastasization
Source: Mol Oncol. 2020 Feb 5;14(3):520–38. doi: 10.1002/1878-0261.12632 (PMC7053247; doi:10.1002/1878-0261.12632)
Supplement: Supplementary file 1 — Table S1. MiRNAs found to be deregulated at 7 days, by NGS analysis [file MOL2-14-520-s001.pdf]

Supplementary Table 1. MiRNAs found to be deregulated at 7 days, by NGS analysis.

miRNA	Readcounts (control)	Readcounts (4T1)	Foldchange
mmu-miR-182-5p	275,538	743,2185	2,6973
mmu-miR-375-3p	219,5051	1062,712	4,8414
mmu-miR-200b-3p	33,6712	14,95501	0,4441
mmu-miR-582-3p	14,3938	52,56911	3,6522
mmu-miR-204-5p	8,7391	18,12728	2,0743
mmu-miR-21a-3p	17,9922	38,52047	2,141
mmu-miR-183-5p	8,7391	28,09728	3,2151
