# Supplementary material for: Downregulation of circulating miR 802‐5p and miR 194‐5p and upregulation of brain MEF2C along breast cancer brain metastasization
Source: Mol Oncol. 2020 Feb 5;14(3):520–38. doi: 10.1002/1878-0261.12632 (PMC7053247; doi:10.1002/1878-0261.12632)
Supplement: Supplementary file 2 — Table S2. MiRNAs found to be deregulated at 10 days, by NGS analysis. [file MOL2-14-520-s002.pdf]

Supplementary Table 2. MiRNAs found to be deregulated at 10 days, by NGS analysis.

miRNA	Readcounts (control)	Readcounts(4T1)	FoldChange
mmu-miR-133a-3p	2067,333238	5169,2123	2,5004
mmu-miR-29a-3p	358,8993044	753,1303	2,0984
mmu-miR-182-5p	446,1271502	2276,1581	5,102
mmu-miR-328-3p	268,3421516	570,0875	2,1245
mmu-miR-146b-5p	469,7652306	1370,725	2,9179
mmu-miR-375-3p	285,9874791	645,5402	2,2572
mmu-miR-423-3p	112,5305797	250,1119	2,2226
mmu-miR-223-3p	120,8538474	444,3329	3,6766
mmu-miR-34c-5p	44,11331896	106,1928	2,4073
mmu-miR-205-5p	65,92028041	234,7419	3,561
mmu-miR-214-3p	98,5474899	41,9182	0,4254
mmu-miR-125b-1-3p	33,95893233	177,4537	5,2255
mmu-miR-132-3p	60,26045835	125,7546	2,0869
mmu-miR-429-3p	31,62841737	68,4664	2,1647
mmu-miR-31-5p	44,61271502	107,59	2,4116
mmu-miR-133b-3p	28,46557563	74,7541	2,6261
mmu-miR-582-3p	19,64291184	55,8909	2,8453
mmu-miR-1843b-5p	26,80092208	74,0555	2,7632
mmu-miR-1843a-5p|mmu-miR-1843b-5p	20,30877326	60,0827	2,9585
mmu-miR-335-5p	25,96859531	54,4937	2,0984
mmu-miR-204-5p	17,31239687	61,48	3,5512
mmu-miR-28a-5p	13,31722837	27,9455	2,0984
mmu-miR-155-5p	19,30998	40,5209	2,0984
mmu-miR-21a-3p	19,64291	58,6855	2,9876
mmu-miR-365-3p	12,65137	5,5891	0,4418
mmu-miR-183-5p	18,97705	79,6446	4,1969
mmu-miR-664-5p	10,32085	29,3427	2,8431
mmu-miR-330-3p	16,3136	36,3291	2,2269
mmu-miR-378a-5p	13,31723	29,3427	2,2034
mmu-miR-431-5p	9,654991	2,7945	0,2894
mmu-miR-223-5p	11,31964	39,1237	3,4563
mmu-miR-339-3p	16,3136	33,5346	2,0556
mmu-miR-301b-3p	10,32085	23,7536	2,3015
mmu-miR-345-3p	12,65137	26,5482	2,0984
mmu-miR-3057-5p	15,98067	32,1373	2,011
mmu-miR-212-3p	5,992753	27,9455	4,6632
mmu-miR-30c-1-3p	6,658614	27,9455	4,1969
mmu-miR-1843a-3p	5,326891	22,3564	4,1969
