# Supplementary material for: Downregulation of circulating miR 802‐5p and miR 194‐5p and upregulation of brain MEF2C along breast cancer brain metastasization
Source: Mol Oncol. 2020 Feb 5;14(3):520–38. doi: 10.1002/1878-0261.12632 (PMC7053247; doi:10.1002/1878-0261.12632)
Supplement: Supplementary file 3 — Table S3. Results of the target prediction for miR‐194‐5p using Target Scan v.7.2 and Diana Tool MicroT‐CDS v.5.0. [file MOL2-14-520-s003.pdf]

Supplementary Table 3. Results of the Target prediction for miR-194-5p using Target Scan v.7.2 and Diana Tool MicroT-CDS v.5.0.

Target Gene	Cumulative weighted context++ score	Total context++ score	Aggregate PCT	MiTG	Target Gene	Cumulativ e weighted context++ score	Total context++ score	Aggregate PCT	MiTG
TRIM23	-0.5	-0.51	0.32	0.981011	AKT2	-0.15	-0.15	0.53	0.773286
TMED5	-0.49	-0.73	0.33	0.831892	ARHGAP24	-0.15	-0.18	0.55	0.796644
SLC10A7	-0.49	-0.7	0.14	0.920473	LPHN2	-0.15	-0.15	0.55	0.841762
ERGIC2	-0.46	-0.46	0.55	0.999352	ERBB4	-0.15	-0.18	0.55	0.903769
TEFM	-0.45	-0.47	< 0.1	0.982956	HIAT1	-0.15	-0.2	0.42	0.864159
STAT1	-0.45	-0.55	< 0.1	0.762706	AMD1	-0.14	-0.32	0.18	0.738216
RAP2B	-0.44	-0.73	0.79	0.813895	CAMK2G	-0.14	-0.14	0.55	0.835199
MID1IP1	-0.43	-0.43	0.33	0.963489	TRIP12	-0.14	-0.2	0.46	0.974147
NUDC	-0.42	-0.52	0.55	0.975467	CHD6	-0.14	-0.14	0.43	0.727125
PAIP2	-0.4	-0.4	0.55	0.954517	PSME3	-0.14	-0.14	0.34	0.720207
ATP6V1H	-0.4	-0.4	0.2	0.715332	RHEB	-0.14	-0.31	0.55	0.855686
SLC7A5	-0.39	-0.42	0.41	0.914903	DNMT3A	-0.14	-0.29	0.79	0.938058
SLK	-0.38	-0.39	0.31	0.981054	DAAM1	-0.14	-0.19	0.3	0.875616
PTPLB	-0.37	-0.37	< 0.1	0.816621	PAFAH1B1	-0.14	-0.15	0.55	0.897828
FAM107B	-0.36	-0.65	< 0.1	0.804205	SGPP2	-0.14	-0.22	< 0.1	0.765868
SNAP91	-0.36	-0.36	0.13	0.872915	SYVN1	-0.13	-0.14	0.55	0.859452
GRHL3	-0.35	-0.35	0.16	0.891536	ACHE	-0.13	-0.39	0.17	0.973065
SLTM	-0.35	-0.36	0.39	0.999316	ELF2	-0.13	-0.13	0.55	0.796334
RSBN1L	-0.35	-0.41	0.57	0.992971	OTP	-0.12	-0.12	0.55	0.710157
SEPHS1	-0.33	-0.37	0.57	0.994116	LIN28B	-0.12	-0.12	< 0.1	0.713877
SETD8	-0.33	-0.33	0.49	0.899924	PVRL4	-0.12	-0.12	< 0.1	0.722846
GOT2	-0.31	-0.43	0.46	0.999811	SETD5	-0.12	-0.2	0.47	0.998517
RFX6	-0.3	-0.3	0.27	0.943858	SNX1	-0.11	-0.16	0.69	0.781367
DUSP9	-0.29	-0.29	0.44	0.733478	PPARGC1A	-0.11	-0.11	0.55	0.811809
DCUN1D5	-0.29	-0.4	0.55	0.908211	OPCML	-0.11	-0.11	0.55	0.87958
TSPAN7	-0.29	-0.29	< 0.1	0.778829	ARID4A	-0.11	-0.11	0.55	0.8312
ARID2	-0.28	-0.28	0.4	0.82587	ITGB6	-0.11	-0.11	< 0.1	0.838143
ARF4	-0.28	-0.28	0.44	0.834818	BICD2	-0.1	-0.26	< 0.1	0.933151
RAB6B	-0.28	-0.28	0.64	0.953928	ONECUT2	-0.1	-0.11	0.62	0.930996
HNF1B	-0.28	-0.28	0.42	0.749551	SATB1	-0.1	-0.13	0.55	0.767405
FBXW7	-0.27	-0.27	0.48	0.990766	ASAP1	-0.1	-0.1	0.39	0.990011
PAK2	-0.27	-0.27	0.14	0.73961	CHD8	-0.1	-0.1	0.26	0.830213
GMFB	-0.27	-0.29	0.26	0.734658	SLC12A6	-0.09	-0.17	0.51	0.857187
KLF7	-0.26	-0.27	0.55	0.952678	MEX3A	-0.09	-0.11	0.28	0.787349
ACBD3	-0.26	-0.27	< 0.1	0.894665	SEMA6A	-0.09	-0.09	0.54	0.714767
ARHGAP21	-0.26	-0.26	0.29	0.976948	HMG20A	-0.08	-0.14	0.35	0.799471
DACH1	-0.26	-0.28	0.22	0.998087	PRKD3	-0.08	-0.33	0.55	0.894082
LPIN2	-0.25	-0.26	0.55	0.891814	RSBN1	-0.08	-0.08	< 0.1	0.733451
PHYHIPL	-0.25	-0.39	< 0.1	0.840235	CLCN5	-0.08	-0.16	0.46	0.852687
FKBP6	-0.25	-0.25	< 0.1	0.817262	MGAT4A	-0.08	-0.17	0.55	0.906412
PRSS53	-0.25	-0.25	0.54	0.928544	RFX3	-0.08	-0.26	0.37	0.904627
HBEGF	-0.25	-0.66	0.69	0.999341	E2F3	-0.08	-0.08	0.54	0.813987
PFN2	-0.23	-0.24	0.55	0.882766	PITPNM2	-0.08	-0.1	0.14	0.895725
SGCE	-0.23	-0.23	0.55	0.851101	TNPO1	-0.07	-0.11	0.55	0.78671
HNRNPA0	-0.23	-0.23	0.55	0.780024	OTUD4	-0.07	-0.07	< 0.1	0.847282
EXOC5	-0.23	-0.23	0.46	0.751349	ADAM12	-0.07	-0.08	< 0.1	0.73022
ELMSAN1	-0.23	-0.23	0.56	0.996329	STAT5B	-0.07	-0.17	0.55	0.872417
FMR1	-0.23	-0.34	< 0.1	0.994802	PPP3R1	-0.07	-0.16	0.23	0.758001
UBE2V2	-0.22	-0.54	0.55	0.787566	SDAD1	-0.06	-0.13	0.55	0.772853
VAPA	-0.22	-0.84	0.69	0.929783	FBRS	-0.06	-0.06	< 0.1	0.899171
FOXA1	-0.22	-0.22	0.36	0.816252	NUCKS1	-0.06	-0.2	0.55	0.714046
NRP1	-0.22	-0.27	0.55	0.894971	LSAMP	-0.06	-0.23	0.43	0.85762
TLN2	-0.22	-0.22	0.56	0.739586	OSBPL8	-0.05	-0.46	0.47	0.87202
SP3	-0.22	-0.22	0.55	0.719156	PTPRD	-0.05	-0.1	0.55	0.954903
KCMF1	-0.21	-0.21	0.55	0.842746	PPFIBP1	-0.05	-0.09	< 0.1	0.845036

EIF4G2	-0.2	-0.2	0.54	0.816995
ENOX1	-0.2	-0.39	0.38	0.999234
NETO1	-0.2	-0.33	0.76	0.936017
SALL1	-0.2	-0.54	0.46	0.998889
REV3L	-0.2	-0.2	0.54	0.981759
TMED9	-0.2	-0.4	0.55	0.944431
DUSP10	-0.19	-0.19	0.55	0.734887
KMT2C	-0.19	-0.19	0.61	0.864119
DR1	-0.19	-0.25	0.55	0.773841
PPP2R2C	-0.19	-0.19	0.5	0.812456
SET	-0.19	-0.19	0.55	0.83123
PRKAR1A	-0.19	-0.26	0.53	0.812713
BCLAF1	-0.19	-0.34	0.55	0.881355
TTC7B	-0.18	-0.3	0.33	0.735821
WAPAL	-0.18	-0.22	0.33	0.9626
MEIS2	-0.18	-0.18	0.43	0.944928
SLC30A1	-0.18	-0.2	0.2	0.870839
COL4A3BP	-0.18	-0.29	0.43	0.92011
CHD4	-0.18	-0.18	0.43	0.889681
CRK	-0.18	-0.18	< 0.1	0.856811
SDC4	-0.18	-0.32	0.52	0.787513
PHF21A	-0.17	-0.17	0.55	0.744362
CDH2	-0.17	-0.17	0.55	0.796689
TCF7L2	-0.17	-0.17	0.55	0.761728
LHX6	-0.17	-0.17	0.55	0.801319
DHX15	-0.17	-0.17	0.55	0.808113
CEP170	-0.17	-0.17	0.14	0.945352
NRIP1	-0.16	-0.16	0.55	0.820419
MEF2C	-0.16	-0.27	0.55	0.741349
ENAH	-0.16	-0.18	0.55	0.863833
TRPS1	-0.05	-0.1	0.51	0.998299
JMJD1C	-0.05	-0.05	0.55	0.845181
ZFHX4	-0.05	-0.05	0.46	0.94843
RUNX3	-0.05	-0.09	< 0.1	0.889468
CCR10	-0.05	-0.36	0.4	0.743165
MAP2	-0.04	-0.06	0.35	0.964614
NAA50	-0.04	-0.32	0.57	0.979536
ITPKB	-0.04	-0.04	0.38	0.982141
DMD	-0.03	-0.2	0.51	0.826378
CHD1	-0.03	-0.17	0.49	0.901686
SOX6	-0.03	-0.09	0.52	0.889379
SMURF1	-0.03	-0.11	0.55	0.915273
SUFU	-0.02	-0.04	< 0.1	0.787976
CEP350	-0.02	-0.02	< 0.1	0.724434
SLC30A10	-0.02	-0.12	0.55	0.875189
BIRC6	-0.02	-0.03	< 0.1	0.803671
CAPZB	-0.02	-0.18	0.55	0.708462
NFAT5	-0.02	-0.04	0.53	0.913664
UBR5	-0.01	-0.12	0.51	0.774026
GIGYF1	-0.01	-0.01	< 0.1	0.900373
PDHB	-0.01	-0.27	0.64	0.721324
CADM1	0	-0.24	0.9	0.972289
LRRC4C	0	-0.28	0.53	0.908255
ZBTB20	0	-0.2	0.61	0.939419
TSC22D2	0	-0.08	0.55	0.793327
NCL	0	-0.27	0.54	0.707306
SUMO2	0	-0.53	0.57	0.999546
RABAC1	0	-0.76	0.22	0.961871
UBE2D3	0	-0.3	< 0.1	0.913441
