# Supplementary material for: Downregulation of circulating miR 802‐5p and miR 194‐5p and upregulation of brain MEF2C along breast cancer brain metastasization
Source: Mol Oncol. 2020 Feb 5;14(3):520–38. doi: 10.1002/1878-0261.12632 (PMC7053247; doi:10.1002/1878-0261.12632)
Supplement: Supplementary file 4 — Table S4. Results of the target prediction for miR‐802‐5p using TargetScan v.7.2 and diana tools MicroT‐CDS v.5.0. [file MOL2-14-520-s004.pdf]

Supplementary Table 4. Results of the target prediction for miR-802-5p using TargetScan v.7.2 and Diana tools MicroT-CDS v.5.0.

Target Gene	Cumulative weighted context++ score	Total context++ score	Aggregate PCT	MiTG	Target Gene	Cumulative weighted context++ score	Total context++ score	Aggregate PCT	MiTG
TMED9	-0.95	-0.96	0.7	0.999909	ZMYM2	-0.13	-0.2	< 0.1	0.952878
SREK1	-0.66	-0.76	0.42	0.985192	FAT1	-0.12	-0.15	0.7	0.981068
FOXJ3	-0.55	-0.55	0.56	0.901663	MBNL1	-0.12	-0.12	0.39	0.801453
KHDRBS1	-0.52	-0.52	0.32	0.999481	LIN54	-0.12	-0.12	< 0.1	0.782849
PSMD2	-0.52	-0.52	< 0.1	0.991373	CDV3	-0.12	-0.15	0.39	0.766561
SCAMP1	-0.47	-0.48	0.48	0.932605	DSCAM	-0.11	-0.11	0.57	0.934438
HSPD1	-0.45	-0.45	0.56	0.875645	EVI5	-0.11	-0.11	0.55	0.775588
SLC2A3	-0.44	-0.44	0.3	0.891992	MEX3B	-0.11	-0.11	0.43	0.781921
MSI1	-0.43	-0.43	0.67	0.856616	HAPLN1	-0.11	-0.11	0.51	0.781952
SCG2	-0.43	-0.43	0.43	0.972459	GABPA	-0.1	-0.1	0.34	0.815857
HNF1B	-0.42	-0.42	0.16	0.999886	DEPDC1B	-0.09	-0.1	0.28	0.755464
XKRX	-0.41	-0.41	< 0.1	0.838433	ZFYVE9	-0.09	-0.22	0.55	0.78931
DDX4	-0.39	-0.39	0.24	0.791042	PRICKLE2	-0.09	-0.09	0.51	0.865375
SMTNL2	-0.38	-0.38	0.35	0.887651	SLC9A6	-0.09	-0.09	0.13	0.742503
RHOA	-0.38	-0.38	0.34	0.817546	NR3C2	-0.09	-0.09	0.32	0.70552
RAN	-0.37	-0.43	0.55	0.922937	GNA13	-0.09	-0.12	0.54	0.854533
RAPGEF4	-0.37	-0.37	0.26	0.984161	ZMYND11	-0.08	-0.1	0.36	0.785629
ARID2	-0.36	-0.36	0.45	0.999041	YWHAE	-0.08	-0.09	0.49	0.927791
SDC4	-0.34	-0.56	0.48	0.981052	CLCN3	-0.08	-0.09	0.51	0.743844
UBE2M	-0.33	-0.33	0.22	0.913111	JMJD1C	-0.08	-0.1	0.46	0.753716
NKRF	-0.32	-0.33	0.53	0.792197	PANK1	-0.08	-0.14	0.56	0.701887
PAFAH1B1	-0.32	-0.45	0.25	0.737278	MYLIP	-0.07	-0.08	0.43	0.797326
CDH11	-0.3	-0.45	0.55	0.858018	PNPLA8	-0.07	-0.14	0.35	0.753913
KLF7	-0.29	-0.3	0.56	0.995724	CACNA1I	-0.07	-0.07	0.55	0.757567
SLC35F1	-0.29	-0.29	0.13	0.907496	DCUN1D3	-0.07	-0.07	0.53	0.77476
CSDE1	-0.28	-0.28	0.57	0.986691	EPM2AIP1	-0.07	-0.17	0.36	0.816662
CDK19	-0.27	-0.37	0.23	0.841852	PEAK1	-0.07	-0.08	0.53	0.70174
MBNL2	-0.27	-0.27	< 0.1	0.965317	SLC33A1	-0.07	-0.15	0.37	0.750557
MIER3	-0.26	-0.3	0.36	0.82333	EDEM3	-0.07	-0.07	0.37	0.75158
STX16	-0.25	-0.35	0.24	0.96191	HECW2	-0.06	-0.09	0.32	0.863959
TBC1D23	-0.25	-0.27	0.23	0.951752	ONECUT2	-0.05	-0.05	0.33	0.707787
TMEM25	-0.24	-0.24	0.18	0.703531	ATXN1	-0.05	-0.06	0.47	0.818501
GATA4	-0.23	-0.23	< 0.1	0.876993	ABAT	-0.04	-0.09	0.55	0.809815
AGFG1	-0.23	-0.23	0.56	0.897033	FZD5	-0.04	-0.25	0.54	0.834818
PPP3CA	-0.22	-0.22	0.38	0.934698	RAPGEF6	-0.04	-0.04	0.54	0.839526
ATP6V1C1	-0.22	-0.23	0.2	0.786791	NDUFB6	-0.03	-0.28	0.42	0.882873
TSHZ3	-0.21	-0.21	0.41	0.939905	MAP3K2	-0.03	-0.18	0.17	0.903692
SLC2A1	-0.2	-0.2	0.39	0.755085	MEF2C	-0.03	-0.09	0.71	0.952272
USP44	-0.19	-0.19	ORF	0.735664	TET3	-0.03	-0.03	0.2	0.732387
MATR3	-0.19	-0.28	0.21	0.9436	ASAP1	-0.03	-0.03	< 0.1	0.869298
TRAF3	-0.18	-0.24	0.42	0.722446	KMT2A	-0.03	-0.03	0.27	0.875507
TCF4	-0.18	-0.22	0.19	0.975189	PPP2CA	-0.03	-0.63	0.56	0.949455
CPEB3	-0.18	-0.18	0.43	0.824353	ATF2	-0.03	-0.03	< 0.1	0.986103
SOX6	-0.17	-0.32	0.41	0.927457	NSD1	-0.03	-0.09	0.44	0.852003
RORA	-0.16	-0.19	0.33	0.816545	KDM5A	-0.02	-0.03	0.38	0.723192
PCSK5	-0.16	-0.16	0.4	0.840574	MYO6	-0.02	-0.18	0.13	0.742437
LRRTM3	-0.16	-0.16	0.53	0.978788	HIPK1	-0.02	-0.02	0.58	0.922359
SLC25A53	-0.16	-0.33	< 0.1	0.801373	SMARCE1	-0.02	-0.11	0.47	0.802616
ZFHX4	-0.15	-0.16	0.49	0.997019	BRD3	-0.02	-0.23	0.17	0.994458
IQSEC1	-0.15	-0.25	0.54	0.745638	MED13	-0.02	-0.08	< 0.1	0.960335
SOAT1	-0.15	-0.15	0.23	0.740805	PIP4K2B	-0.01	-0.23	0.54	0.887162
HIAT1	-0.15	-0.28	0.55	0.841361	ERMP1	-0.01	-0.23	0.16	0.774486
ARMC8	-0.15	-0.15	0.45	0.74319	UFD1L	-0.01	-0.28	0.55	0.717176
NEGR1	-0.14	-0.17	0.37	0.813117	DCTN4	-0.01	-0.21	0.26	0.757104
TAB3	-0.14	-0.15	0.19	0.780968	FOXP1	-0.01	-0.08	0.47	0.903238
TOB1	-0.14	-0.14	0.48	0.895328	ARHGEF12	0	-0.01	0.55	0.915539

ZFHX3	-0.14	-0.18	0.47	0.993017	IMMT	0	-0.08	0.55	0.734088
NMT2	-0.13	-0.39	0.55	0.997604	TLK2	0	-0.13	0.12	0.847124
SETD2	-0.13	-0.14	0.45	0.924397	NFIB	0	-0.16	0.49	0.991528

ORF, Open Reading Frame
