# Supplementary material for: Downregulation of circulating miR 802‐5p and miR 194‐5p and upregulation of brain MEF2C along breast cancer brain metastasization
Source: Mol Oncol. 2020 Feb 5;14(3):520–38. doi: 10.1002/1878-0261.12632 (PMC7053247; doi:10.1002/1878-0261.12632)
Supplement: Supplementary file 5 — Table S5. Results of the target prediction for miR‐17‐3p using TargetScan v.7.2 and diana tools MicroT‐CDS v.5.0. [file MOL2-14-520-s005.pdf]

Supplementary Table 5. Results of the target prediction for miR-17-3p using TargetScan v.7.2 and Diana Tools MicroT-CDS v.5.0.

Target Gene	Cumulative weighted context++ score	Total context++ score	Aggregate PCT	MiTG	Target Gene	Cumulative weighted context++ score	Total context++ score	Aggregate PCT	MiTG
YIPF5	-0.69	-0.69	N/A	0.8639967	KCND3	-0.12	-0.12	N/A	0.8582322
GREB1L	-0.68	-0.68	N/A	0.7341568	CTNND1	-0.12	-0.29	N/A	0.9718884
BBX	-0.63	-0.67	N/A	0.9247143	CACNA1D	-0.12	-0.12	N/A	0.7238547
LYVE1	-0.62	-0.86	N/A	0.7751044	FRK	-0.12	-0.26	N/A	0.7194361
GYPC	-0.61	-0.61	N/A	0.9633201	CYTH1	-0.12	-0.12	N/A	0.7684528
RECQL4	-0.61	-0.61	N/A	0.7522649	RORA	-0.12	-0.14	N/A	0.7170271
SMARCA5	-0.55	-0.66	N/A	0.7654935	BRWD3	-0.12	-0.15	N/A	0.9109003
TSC1	-0.54	-0.6	N/A	0.8136672	SORCS2	-0.11	-0.15	N/A	0.7746416
NEUROG1	-0.54	-0.54	N/A	0.7918253	DACH1	-0.11	-0.11	N/A	0.7437861
SLC40A1	-0.52	-0.65	N/A	0.7977602	VSIG10	-0.11	-0.33	N/A	0.7185098
ATF1	-0.5	-0.59	N/A	0.7132483	STOX2	-0.11	-0.12	N/A	0.8190927
ATG10	-0.48	-0.61	N/A	0.775864	KIF5C	-0.11	-0.25	N/A	0.9364542
PPP2CA	-0.48	-0.48	N/A	0.8782823	PCDHA10	-0.11	-0.11	N/A	0.7241824
RAP2A	-0.47	-0.49	N/A	0.7715476	KCNA1	-0.11	-0.11	N/A	0.8038043
VPS36	-0.44	-0.46	N/A	0.8148425	LRRC14	-0.11	-0.13	N/A	0.7518062
RIT1	-0.43	-0.55	N/A	0.7565781	CHD7	-0.11	-0.12	N/A	0.7767347
WIBG	-0.42	-0.42	N/A	0.7737177	PTPN2	-0.11	-0.18	N/A	0.7482413
SMEK1	-0.41	-0.41	N/A	0.974589	COPS2	-0.11	-0.11	N/A	0.7320244
NRF1	-0.4	-0.4	N/A	0.7572689	ARID4A	-0.11	-0.11	N/A	0.7611902
ELTD1	-0.4	-0.4	N/A	0.7401522	PSD3	-0.11	-0.12	N/A	0.7071386
EBF1	-0.39	-0.39	N/A	0.9139082	BTF3	-0.11	-0.26	N/A	0.7688861
RAB21	-0.39	-0.39	N/A	0.9853384	KBTBD2	-0.11	-0.11	N/A	0.7413645
OBSCN	-0.38	-0.38	N/A	0.7413025	ZFHX2	-0.1	-0.1	N/A	0.9025715
RAP2C	-0.38	-0.53	N/A	0.9682747	LPIN2	-0.1	-0.1	N/A	0.7396211
FAM169A	-0.37	-0.4	N/A	0.8879096	PLEKHG4	-0.1	-0.1	N/A	0.7570234
RAB8B	-0.35	-0.36	N/A	0.9671688	COL12A1	-0.1	-0.56	N/A	0.9932888
IPO5	-0.34	-0.4	N/A	0.8010351	IMPDH1	-0.1	-0.1	N/A	0.7048521
SMIM14	-0.34	-0.43	N/A	0.9275585	SPRED2	-0.1	-0.11	N/A	0.8108673
PPP6R1	-0.34	-0.41	N/A	0.7903077	KRAS	-0.1	-0.29	N/A	0.843668
USP46	-0.34	-0.38	N/A	0.8400438	GALNTL6	-0.1	-0.1	N/A	0.7798187
MLXIP	-0.34	-0.41	N/A	0.7709477	CAPN7	-0.09	-0.45	N/A	0.8712637
ERGIC2	-0.34	-0.37	N/A	0.7976461	TAB1	-0.09	-0.1	N/A	0.7096626
MED13L	-0.34	-0.51	N/A	0.9051891	PELI2	-0.09	-0.11	N/A	0.7375602
PPM1F	-0.33	-0.36	N/A	0.743312	AGFG1	-0.09	-0.1	N/A	0.7125768
YY2	-0.32	-0.32	N/A	0.7481928	RCOR1	-0.09	-0.14	N/A	0.7636254
EYA3	-0.31	-0.31	N/A	0.8353457	SOX11	-0.09	-0.09	N/A	0.7813589
FGFR1OP	-0.31	-0.59	N/A	0.9122515	SRSF10	-0.09	-0.1	N/A	0.8544207
TES	-0.3	-0.3	N/A	0.8357008	TNRC6B	-0.09	-0.11	N/A	0.7632446
HP1BP3	-0.3	-0.32	N/A	0.719626	POU6F1	-0.09	-0.09	N/A	0.7309603
AP2A2	-0.3	-0.31	N/A	0.7513344	DPYSL2	-0.08	-0.08	N/A	0.8330467
ARHGEF40	-0.3	-0.32	N/A	0.7442224	SMURF1	-0.08	-0.35	N/A	0.7056469
FAM45A	-0.3	-0.3	N/A	0.7029161	TBC1D30	-0.08	-0.41	N/A	0.7948308
AGO4	-0.29	-0.37	N/A	0.7657916	RREB1	-0.08	-0.08	N/A	0.7292753
THSD4	-0.29	-0.46	N/A	0.8286226	ZFR	-0.08	-0.18	N/A	0.7391855
APAF1	-0.29	-0.29	N/A	0.847014	DCLK1	-0.08	-0.17	N/A	0.823037
GABPB2	-0.29	-0.38	N/A	0.7665388	JARID2	-0.08	-0.08	N/A	0.891243
FKBP5	-0.28	-0.28	N/A	0.7943886	BAI1	-0.08	-0.08	N/A	0.7712396
SLC35B2	-0.28	-0.28	N/A	0.7278006	RC3H1	-0.08	-0.1	N/A	0.7541873
STXBP1	-0.28	-0.39	N/A	0.724675	CBLL1	-0.08	-0.17	N/A	0.7015517
TDRKH	-0.28	-0.29	N/A	0.7896945	POU2F1	-0.08	-0.08	N/A	0.8009264
AGGF1	-0.28	-0.29	N/A	0.8552118	FAF1	-0.08	-0.18	N/A	0.8756497
NAMPT	-0.28	-0.29	N/A	0.742962	TAB2	-0.07	-0.07	N/A	0.7021732
TNP2	-0.27	-0.27	N/A	0.7715569	SMG6	-0.07	-0.07	N/A	0.9076736
CDCP1	-0.27	-0.36	N/A	0.7315804	TMX4	-0.07	-0.1	N/A	0.845652
GKAP1	-0.27	-0.27	N/A	0.8668482	ZNRF2	-0.07	-0.35	N/A	0.7995211
PIGT	-0.27	-0.27	N/A	0.7359581	GPBP1	-0.07	-0.18	N/A	0.9068783
OSBPL5	-0.27	-0.27	N/A	0.7078302	FAM219B	-0.07	-0.08	N/A	0.7033691

MACROD2	-0.26	-0.26	N/A	0.921538	CAMSAP1	-0.06	-0.1	N/A	0.7673285
PPARGC1A	-0.26	-0.36	N/A	0.9174552	TEAD1	-0.06	-0.06	N/A	0.7477532
TSHZ3	-0.25	-0.3	N/A	0.7732079	LRFN1	-0.06	-0.06	N/A	0.755691
PDE7A	-0.25	-0.76	N/A	0.80876	CREBL2	-0.06	-0.24	N/A	0.724639
RAP1GAP2	-0.24	-0.26	N/A	0.7157019	RBMS3	-0.06	-0.1	N/A	0.7094809
AEBP2	-0.24	-0.24	N/A	0.8395793	EML5	-0.06	-0.07	N/A	0.7319119
DCHS1	-0.24	-0.24	N/A	0.9728169	TMOD1	-0.06	-0.15	N/A	0.7035133
B3GAT2	-0.24	-0.25	N/A	0.7756316	ARMC8	-0.06	-0.06	N/A	0.8213741
RAD52	-0.23	-0.23	N/A	0.7541637	AGO3	-0.05	-0.2	N/A	0.8588968
GTF2H1	-0.23	-0.27	N/A	0.7152589	SGIP1	-0.05	-0.1	N/A	0.7728853
MBNL1	-0.23	-0.24	N/A	0.9296103	MAML1	-0.05	-0.05	N/A	0.7258857
CALU	-0.23	-0.32	N/A	0.7426807	CSDE1	-0.05	-0.05	N/A	0.9278072
YWHAG	-0.23	-0.23	N/A	0.9609502	IMPG2	-0.05	-0.05	N/A	0.8558744
KAT7	-0.22	-0.23	N/A	0.7890975	HOMEZ	-0.05	-0.13	N/A	0.7693477
SYNCRIP	-0.22	-0.25	N/A	0.9923945	RBM46	-0.04	-0.11	N/A	0.7221442
RAB6A	-0.22	-0.22	N/A	0.8262028	IL1R1	-0.04	-0.04	N/A	0.7099776
CAMK2B	-0.21	-0.21	N/A	0.7154958	TBCEL	-0.04	-0.07	N/A	0.8736542
MARCKS	-0.21	-0.22	N/A	0.7169878	GFPT1	-0.04	-0.05	N/A	0.7592268
RAB11A	-0.21	-0.38	N/A	0.7902463	MAP9	-0.04	-0.16	N/A	0.790203
SMG7	-0.21	-0.27	N/A	0.7835293	RALGAPA2	-0.04	-0.04	N/A	0.7473901
PTPRZ1	-0.21	-0.21	N/A	0.7423449	MGAT4A	-0.04	-0.22	N/A	0.9033762
PSIP1	-0.2	-0.21	N/A	0.7606127	NFAT5	-0.03	-0.06	N/A	0.9814471
BCORL1	-0.2	-0.2	N/A	0.7692001	LHX9	-0.03	-0.04	N/A	0.8410458
RPL22	-0.2	-0.2	N/A	0.7291515	SLK	-0.03	-0.05	N/A	0.7440095
VASH2	-0.2	-0.2	N/A	0.7234781	PUM2	-0.03	-0.08	N/A	0.9319397
RHOT1	-0.2	-0.2	N/A	0.8910875	SETD5	-0.03	-0.13	N/A	0.8279016
HMGA2	-0.2	-0.2	N/A	0.7020276	TCEB1	-0.03	-0.27	N/A	0.8822742
MAPK10	-0.2	-0.2	N/A	0.7611201	GMPS	-0.03	-0.03	N/A	0.7838124
CTR9	-0.2	-0.2	N/A	0.8015285	PLXNA4	-0.03	-0.04	N/A	0.8199791
B3GNT5	-0.19	-0.19	N/A	0.8102692	WIPF2	-0.03	-0.11	N/A	0.7495976
MISP	-0.19	-0.19	N/A	0.7168311	GAREM	-0.03	-0.04	N/A	0.7308396
KANSL3	-0.19	-0.19	N/A	0.757107	REST	-0.03	-0.13	N/A	0.7692951
TSHZ1	-0.18	-0.19	N/A	0.9040266	SMCR8	-0.03	-0.03	N/A	0.7003351
FAM188B	-0.18	-0.18	N/A	0.7799816	SLC24A4	-0.03	-0.14	N/A	0.7116228
TSTD2	-0.18	-0.18	N/A	0.7041387	IKBKG	-0.03	-0.03	N/A	0.7392743
RMND5A	-0.18	-0.24	N/A	0.7751476	RASAL2	-0.03	-0.06	N/A	0.8417864
RPTOR	-0.18	-0.26	N/A	0.7823752	TANC2	-0.03	-0.03	N/A	0.733032
YWHAQ	-0.18	-0.18	N/A	0.8980721	TFDP2	-0.03	-0.16	N/A	0.7591272
SYNJ1	-0.17	-0.19	N/A	0.9178583	BTRC	-0.03	-0.09	N/A	0.809649
ITGB8	-0.17	-0.24	N/A	0.8402185	FAM129A	-0.02	-0.12	N/A	0.7521037
RNF111	-0.17	-0.19	N/A	0.9232311	CBLB	-0.02	-0.12	N/A	0.7158528
CDK17	-0.17	-0.17	N/A	0.8356761	UNC80	-0.02	-0.02	N/A	0.7612192
PICALM	-0.17	-0.17	N/A	0.7723705	SOD2	-0.02	-0.48	N/A	0.8354911
TENM1	-0.17	-0.17	N/A	0.7545008	HIPK3	-0.02	-0.02	N/A	0.7631568
USP9X	-0.16	-0.22	N/A	0.9207345	MDGA1	-0.02	-0.02	N/A	0.7100004
EPC2	-0.16	-0.21	N/A	0.9229993	OTUD7B	-0.02	-0.16	N/A	0.731045
TUFT1	-0.16	-0.16	N/A	0.8137373	SLC31A1	-0.02	-0.17	N/A	0.7307939
CELF1	-0.16	-0.3	N/A	0.7177978	PTPN4	-0.02	-0.11	N/A	0.757717
DDI2	-0.16	-0.4	N/A	0.7438274	VGLL4	-0.02	-0.14	N/A	0.8534877
CCP110	-0.16	-0.17	N/A	0.7457681	FAR2	-0.02	-0.02	N/A	0.7663866
VEZF1	-0.16	-0.37	N/A	0.9733734	MECP2	-0.02	-0.1	N/A	0.7198737
ATF2	-0.16	-0.24	N/A	0.8141578	HNRNPA3	-0.02	-0.18	N/A	0.9380573
SHISA9	-0.16	-0.16	N/A	0.9137113	DOT1L	-0.02	-0.02	N/A	0.7299091
TTLL12	-0.16	-0.39	N/A	0.8916218	TRAF6	-0.02	-0.08	N/A	0.7232861
CTNNB1	-0.16	-0.16	N/A	0.8103862	SGPL1	-0.02	-0.16	N/A	0.7663172
ANKRD50	-0.16	-0.17	N/A	0.8979081	HDAC5	-0.02	-0.1	N/A	0.8508511
CENPF	-0.16	-0.34	N/A	0.8356583	TIA1	-0.01	-0.04	N/A	0.718981
TRIM39	-0.16	-0.16	N/A	0.7548404	KPNA4	-0.01	-0.02	N/A	0.7790231
PRRT2	-0.15	-0.15	N/A	0.7112107	STRBP	-0.01	-0.13	N/A	0.7869967
UBL3	-0.15	-0.16	N/A	0.7474652	HNRNPR	-0.01	-0.28	N/A	0.7999043
SCYL2	-0.15	-0.26	N/A	0.7317213	IVNS1ABP	-0.01	-0.11	N/A	0.8481355
UHMK1	-0.15	-0.19	N/A	0.85175	CREBRF	-0.01	-0.03	N/A	0.8240566
GOPC	-0.15	-0.17	N/A	0.7385807	SLC35E3	-0.01	-0.3	N/A	0.7299878
IGSF3	-0.15	-0.24	N/A	0.7732636	ANK1	-0.01	-0.01	N/A	0.8547282

FZD4	-0.15	-0.15	N/A	0.7488218	EIF5B	-0.01	-0.06	N/A	0.8528483
MORC3	-0.15	-0.46	N/A	0.7188805	CHRNA5	-0.01	-0.33	N/A	0.8558319
CTBP2	-0.15	-0.15	N/A	0.8282033	TNRC6C	-0.01	-0.14	N/A	0.8402122
DDX6	-0.15	-0.17	N/A	0.7822349	SOCS7	-0.01	-0.06	N/A	0.9248643
TIAM2	-0.14	-0.14	N/A	0.750022	RTN4RL1	-0.01	-0.04	N/A	0.7608192
MKLN1	-0.14	-0.22	N/A	0.9384952	PLA2G16	0	-0.33	N/A	0.7162561
SCRN3	-0.14	-0.18	N/A	0.7823007	CASK	0	-0.27	N/A	0.7049347
ZFHX4	-0.14	-0.14	N/A	0.9673644	VEZT	0	-0.33	N/A	0.7882631
SEMA3D	-0.14	-0.17	N/A	0.8522822	CCSER2	0	-0.02	N/A	0.7121685
RAVER2	-0.14	-0.18	N/A	0.7036183	CCDC171	0	-0.03	N/A	0.7822345
AAK1	-0.14	-0.27	N/A	0.7286299	RPL10L	0	-0.61	N/A	0.9360822
ARHGEF38	-0.14	-0.14	N/A	0.7517767	MAP4K2	0	-0.13	N/A	0.8542458
TMEM245	-0.13	-0.14	N/A	0.8749779	KMT2E	0	-0.03	N/A	0.8421474
CHST11	-0.13	-0.16	N/A	0.7320669	TIMD4	0	-0.16	N/A	0.7098387
KPNA3	-0.13	-0.17	N/A	0.8535817	ZBTB20	0	-0.09	N/A	0.8288886
PTEN	-0.13	-0.17	N/A	0.7372004	TG	0	-0.09	N/A	0.9766646
SNX27	-0.13	-0.13	N/A	0.971913	PHIP	0	-0.02	N/A	0.7472064
AP3M1	-0.13	-0.13	N/A	0.8333553	BST1	0	-0.35	N/A	0.7548686
MEIS1	-0.13	-0.13	N/A	0.8291042	CCDC132	0	-0.12	N/A	0.8849029
ABCC1	-0.12	-0.12	N/A	0.8034424					

N/A, Not Applicable
