# Supplementary material for: Downregulation of circulating miR 802‐5p and miR 194‐5p and upregulation of brain MEF2C along breast cancer brain metastasization
Source: Mol Oncol. 2020 Feb 5;14(3):520–38. doi: 10.1002/1878-0261.12632 (PMC7053247; doi:10.1002/1878-0261.12632)
Supplement: Supplementary file 6 — Table S6. Results of the target prediction for miR‐145‐5p using TargetScan v.7.2. and diana tools MicroT‐CDS v.5.0. [file MOL2-14-520-s006.pdf]

Supplementary Table 6. Results of the target prediction for miR-145-5p using TargetScan v.7.2. and Diana Tools MicroT-CDS v.5.0.

Target Gene	Cumulative weighted context++ score	Total context++ score	Aggregate PCT	MiTG	Taget Gene	Cumulative weighted context++ score	Total context++ score	Aggregate PCT	MiTG
FSCN1	-1.19	-1.21	> 0.99	0.990026	SMCR8	-0.19	-0.2	0.35	0.841142
ABRACL	-0.9	-0.91	0.69	0.989389	RBPMS	-0.19	-0.19	0.61	0.914387
FLI1	-0.89	-0.89	> 0.99	0.999025	MPZL1	-0.19	-0.19	0.71	0.703166
SMCP	-0.69	-0.69	0.42	0.723234	ARPC5	-0.19	-0.22	0.55	0.726186
GLIS1	-0.67	-0.67	0.89	0.990084	KLHDC10	-0.18	-0.18	0.68	0.704933
FKBP3	-0.62	-0.62	0.59	0.719159	FOXO1	-0.18	-0.18	0.58	0.700853
YTHDF2	-0.56	-0.56	0.7	0.972194	MAPK4	-0.18	-0.2	< 0.1	0.707789
PPP3CA	-0.56	-0.56	0.92	0.977589	PHACTR2	-0.18	-0.21	0.76	0.953756
KCNA4	-0.56	-0.56	0.6	0.913587	NFE2L1	-0.17	-0.17	0.79	0.878556
MYO5A	-0.53	-0.6	0.96	0.934889	RREB1	-0.17	-0.17	0.74	0.742885
DAB2	-0.53	-0.73	0.93	0.997652	RIMS1	-0.17	-0.17	0.87	0.94063
RTKN	-0.53	-0.53	0.7	0.996007	AKIRIN1	-0.17	-0.19	0.65	0.927232
NTN4	-0.52	-0.52	0.7	0.982168	ABCA1	-0.17	-0.17	0.99	0.960825
SCAMP3	-0.52	-0.62	0.39	0.795711	TBC1D14	-0.17	-0.17	0.33	0.912571
OTX2	-0.51	-0.51	0.57	0.929034	ROCK1	-0.17	-0.19	> 0.99	0.700584
RNF170	-0.51	-0.52	0.85	0.864006	GDNF	-0.17	-0.17	0.58	0.732602
UXS1	-0.5	-0.5	0.78	0.985649	ZFYVE9	-0.16	-0.42	0.93	0.942182
SEMA3A	-0.49	-0.58	0.98	0.999759	ERN1	-0.16	-0.17	0.9	0.957114
MEST	-0.49	-0.6	0.77	0.799471	ARHGAP12	-0.16	-0.16	0.38	0.931137
FAM135A	-0.47	-0.47	0.88	0.79282	ABR	-0.16	-0.16	0.61	0.715615
MPZL2	-0.47	-0.77	0.78	0.983932	CFL2	-0.15	-0.43	0.82	0.818795
ADPGK	-0.47	-0.47	0.78	0.860312	NRAS	-0.15	-0.53	0.87	0.946294
MBTD1	-0.46	-0.46	0.72	0.929347	SSBP3	-0.15	-0.21	0.69	0.755927
CAMSAP2	-0.44	-0.44	0.89	0.988622	KIF21A	-0.15	-0.15	0.53	0.924933
CASZ1	-0.44	-0.44	0.93	0.985079	CDC37L1	-0.15	-0.26	0.9	0.931664
SPSB4	-0.44	-0.44	0.88	0.818235	SPATS2	-0.15	-0.33	0.78	0.951666
UBASH3A	-0.44	-0.44	< 0.1	0.740245	AKAP12	-0.15	-0.18	0.78	0.952811
YES1	-0.43	-0.43	0.98	0.994265	EIF4A2	-0.14	-0.19	0.4	0.738121
SRGAP2	-0.43	-0.49	> 0.99	0.935824	PDGFD	-0.14	-0.43	0.18	0.716554
ACTB	-0.43	-0.45	0.76	0.985066	BACH2	-0.14	-0.14	0.78	0.831347
BTF3L4	-0.43	-0.44	0.5	0.721509	LHFPL2	-0.14	-0.14	0.71	0.76497
SPOP	-0.43	-0.43	0.35	0.96767	FAXC	-0.14	-0.14	0.65	0.834146
GABARAPL2	-0.42	-0.42	0.61	0.838191	MAP3K11	-0.14	-0.14	0.71	0.770841
DAW1	-0.41	-0.41	ORF	0.824445	ONECUT2	-0.14	-0.15	0.62	0.919817
ABHD17B	-0.41	-0.41	0.75	0.982999	CSMD3	-0.14	-0.14	0.57	0.704122
ERG	-0.41	-0.41	0.91	0.904573	NAA40	-0.13	-0.2	0.91	0.780867
MKL2	-0.41	-0.41	> 0.99	0.907962	AP1G1	-0.13	-0.14	0.49	0.897918
LDLRAD3	-0.41	-0.41	0.94	0.987876	DLC1	-0.13	-0.14	0.98	0.790943
CTNNBIP1	-0.41	-0.59	0.9	0.979857	CACHD1	-0.13	-0.13	0.53	0.770038
CAMK1D	-0.4	-0.44	0.99	0.922844	RNF216	-0.13	-0.18	0.78	0.945081
NEDD9	-0.39	-0.39	0.88	0.99835	DENND5B	-0.12	-0.13	0.77	0.776691
PPP4R2	-0.39	-0.39	0.83	0.964532	EFNA3	-0.12	-0.3	0.37	0.809025
CDO1	-0.39	-0.39	0.44	0.926782	FNDC3B	-0.12	-0.2	0.56	0.965382
CCDC25	-0.38	-0.39	0.84	0.947156	PLXNA2	-0.12	-0.12	0.66	0.726211
ELMO1	-0.38	-0.38	0.89	0.94292	PURA	-0.12	-0.23	0.78	0.945279
PAN2	-0.38	-0.38	0.78	0.963907	PLAGL2	-0.12	-0.19	0.67	0.785642
H2AFX	-0.37	-0.37	0.74	0.720457	LRRC16A	-0.12	-0.12	0.42	0.803012
EBF3	-0.37	-0.42	0.86	0.920238	UGCG	-0.12	-0.12	0.58	0.858846
CTNND1	-0.37	-0.37	0.89	0.852615	SLC25A25	-0.11	-0.12	0.65	0.779924
TNFRSF11B	-0.36	-0.36	0.43	0.872078	FNDC3A	-0.11	-0.16	0.55	0.816107
PCSK5	-0.36	-0.36	0.68	0.953307	KLHL15	-0.11	-0.11	0.69	0.724496
ERLIN1	-0.35	-0.36	0.96	0.929362	HNRNPH2	-0.11	-0.71	0.62	0.996289
DUSP6	-0.35	-0.35	0.9	0.954825	ZBTB10	-0.1	-0.15	0.94	0.917429
ZHX2	-0.34	-0.34	0.83	0.829234	TRIO	-0.1	-0.32	0.85	0.930811
CITED2	-0.34	-0.42	0.68	0.8771	RAB14	-0.1	-0.29	0.68	0.90319
GMFB	-0.33	-0.51	0.98	0.829209	BICC1	-0.1	-0.1	0.69	0.796465
NUAK1	-0.33	-0.33	0.99	0.951275	UBN2	-0.1	-0.12	0.86	0.935107

MAGI2	-0.33	-0.33	0.86	0.912266
GTPBP8	-0.32	-0.68	0.44	0.706368
DERL2	-0.32	-0.35	0.75	0.877265
GGT7	-0.31	-0.31	0.29	0.824598
STAM	-0.31	-0.35	0.84	0.723395
SRGAP1	-0.31	-0.35	0.96	0.982833
YTHDC1	-0.3	-0.43	0.94	0.832085
DACH1	-0.3	-0.3	0.76	0.850352
XRN1	-0.3	-0.31	0.7	0.873308
SERINC5	-0.3	-0.32	0.81	0.799004
GRB10	-0.3	-0.3	0.7	0.968735
BBIP1	-0.29	-0.29	ORF	0.72055
PLCE1	-0.29	-0.29	0.88	0.949073
SLC7A8	-0.28	-0.28	0.46	0.741942
OSBPL1A	-0.28	-0.29	0.22	0.862754
NR4A2	-0.28	-0.28	0.31	0.757461
UBA6	-0.27	-0.36	0.82	0.894901
SMAD3	-0.27	-0.35	0.85	0.779252
ANKRD28	-0.27	-0.29	0.89	0.879218
JPH1	-0.27	-0.27	0.66	0.948413
ADAM17	-0.26	-0.28	0.71	0.714059
GLIS3	-0.26	-0.26	0.92	0.984963
MTX3	-0.26	-0.28	0.65	0.956376
PLCL2	-0.26	-0.28	0.56	0.964494
DYRK1A	-0.26	-0.26	0.79	0.836101
SMAD5	-0.26	-0.33	0.83	0.852461
UNC119B	-0.26	-0.26	0.45	0.795006
ANGPT2	-0.26	-0.26	0.91	0.941583
MIER3	-0.25	-0.26	0.72	0.846603
ZBTB46	-0.25	-0.25	0.82	0.944498
PXN	-0.25	-0.25	0.62	0.846161
CYR61	-0.25	-0.25	0.72	0.75683
PTGFR	-0.25	-0.25	0.8	0.941631
TAGLN2	-0.24	-0.24	0.68	0.867969
IPMK	-0.24	-0.24	0.74	0.949723
ERF	-0.24	-0.25	0.59	0.79474
HS6ST1	-0.23	-0.23	0.79	0.70318
ARHGAP24	-0.23	-0.23	0.79	0.71821
CSTF3	-0.23	-0.23	0.55	0.867998
TTC14	-0.23	-0.23	0.41	0.840476
SLITRK4	-0.23	-0.24	0.27	0.86923
AP3S1	-0.23	-0.23	0.46	0.951394
ATXN2	-0.22	-0.23	0.79	0.965054
CPEB1	-0.22	-0.22	0.63	0.788947
EBF1	-0.22	-0.39	0.71	0.847652
LOX	-0.22	-0.38	0.71	0.735396
SNX27	-0.21	-0.21	0.89	0.81629
PAK7	-0.21	-0.21	0.26	0.942968
SP9	-0.2	-0.2	0.46	0.7094
CAMK2D	-0.2	-0.2	0.92	0.840552
IKZF2	-0.2	-0.21	0.58	0.769321
SLC1A2	-0.2	-0.2	0.44	0.777873
REV3L	-0.2	-0.26	0.72	0.995828
DOCK9	-0.2	-0.36	0.59	0.910929
TRIM2	-0.19	-0.19	0.59	0.993785
SOS2	-0.1	-0.1	0.58	0.75894
ACSL4	-0.09	-0.16	0.68	0.802012
POU6F2	-0.09	-0.09	0.56	0.932484
CARF	-0.09	-0.12	0.13	0.924729
ADCYAP1	-0.08	-0.73	0.31	0.946055
MICAL3	-0.08	-0.08	0.97	0.745035
ZDHHC9	-0.08	-0.08	0.45	0.720887
RBPMS2	-0.07	-0.33	0.74	0.928339
MRGBP	-0.07	-0.34	0.54	0.706744
FLNB	-0.07	-0.08	0.75	0.788112
GPHN	-0.07	-0.07	ORF	0.760945
NUFIP2	-0.07	-0.4	0.95	0.950969
ASAP2	-0.07	-0.16	0.78	0.909873
RFX3	-0.07	-0.11	0.64	0.782173
USP31	-0.06	-0.21	0.69	0.988014
ZDHHC14	-0.06	-0.4	0.2	0.738028
MAP3K3	-0.06	-0.06	0.36	0.820314
CLIP1	-0.06	-0.1	0.46	0.971009
PSD3	-0.06	-0.06	0.76	0.937893
SFXN1	-0.06	-0.17	0.63	0.773642
DPYSL2	-0.05	-0.05	0.69	0.795619
RBM20	-0.05	-0.07	0.81	0.778074
ABHD5	-0.05	-0.25	0.52	0.797671
PRR14L	-0.05	-0.05	0.18	0.704938
HOXC11	-0.04	-0.04	ORF	0.83676
KATNBL1	-0.04	-0.4	0.63	0.834385
CAPRIN1	-0.04	-0.12	0.57	0.912714
FAM126A	-0.04	-0.19	0.74	0.982906
NFIB	-0.04	-0.14	0.72	0.96908
ATXN7L3	-0.03	-0.03	0.5	0.801344
ATRX	-0.03	-0.05	0.65	0.731173
PLEKHH1	-0.03	-0.12	0.54	0.917543
ADAM19	-0.03	-0.03	0.7	0.746902
HIC2	-0.03	-0.12	0.96	0.913012
ARHGAP19	-0.03	-0.03	0.36	0.764898
SOX11	-0.03	-0.13	0.68	0.734613
TLN2	-0.02	-0.05	0.44	0.79691
ATXN7L1	-0.02	-0.02	0.54	0.833306
PHF21A	-0.02	-0.08	0.8	0.742215
AMOTL2	-0.02	-0.02	0.38	0.864452
CAPZB	-0.02	-0.23	0.82	0.866677
SOCS7	-0.02	-0.19	0.67	0.947534
MED13	-0.01	-0.16	0.77	0.984545
SEMA6A	-0.01	-0.16	0.36	0.800489
INO80	-0.01	-0.28	0.82	0.745937
SESN3	-0.01	-0.13	0.72	0.761828
PCBP2	-0.01	-0.33	0.45	0.907787
CDH2	0	-0.13	0.73	0.899534
ORC4	0	-0.56	0.91	0.942566
SLC25A11	0	-0.22	0.56	0.948354
EXT1	0	-0.18	0.45	0.801822
ZBTB20	0	-0.4	> 0.99	0.987385
TGFBR2	0	-0.13	0.93	0.971561
INSIG1	0	-0.53	0.78	0.963813

ORF, Open Reading Frame
