# Supplementary material for: Downregulation of circulating miR 802‐5p and miR 194‐5p and upregulation of brain MEF2C along breast cancer brain metastasization
Source: Mol Oncol. 2020 Feb 5;14(3):520–38. doi: 10.1002/1878-0261.12632 (PMC7053247; doi:10.1002/1878-0261.12632)
Supplement: Supplementary file 7 — Table S7. Results of the target prediction for miR‐338‐3p using TargetScan v.7.2 and diana tools MicroT‐CDS v.5.0. [file MOL2-14-520-s007.pdf]

Supplementary Table 7. Results of the target prediction for miR-338-3p using TargetScan v.7.2 an Diana Tools MicroT-CDS v.5.0.

Target Gene	Cumulative weighted context++ score	Total context++ score	Aggregate PCT	MiTG	Target Gene	Cumulative weighted context++ score	Total context+ + score	Aggregate PCT	MiTG
COX4I1	-0.75	-0.75	< 0.1	0.9380724	ACTR2	-0.16	-0.2	< 0.1	0.7900001
ETS1	-0.72	-0.75	0.42	0.9949579	EHF	-0.16	-0.48	0.16	0.7767183
DCAF12	-0.7	-0.7	0.26	0.972957	PCDH1	-0.16	-0.16	< 0.1	0.8347269
PVALB	-0.66	-0.66	< 0.1	0.9860406	UBFD1	-0.16	-0.16	0.31	0.872668
ARHGEF10L	-0.61	-0.62	< 0.1	0.9669559	CBFB	-0.16	-0.19	0.16	0.8237471
CHTOP	-0.57	-0.6	0.26	0.9230866	PPP1R3F	-0.16	-0.16	0.31	0.7460611
FAM89B	-0.56	-0.56	< 0.1	0.9650825	SOX4	-0.15	-0.15	0.31	0.7883954
MTUS1	-0.54	-0.54	0.24	0.9867335	WNK1	-0.15	-0.21	0.42	0.7124502
FKBP1A	-0.54	-0.61	0.3	0.971708	EYA3	-0.15	-0.15	0.28	0.7591097
ZBTB18	-0.54	-0.54	0.49	0.9992805	CPEB3	-0.15	-0.16	0.16	0.7405411
TANGO2	-0.52	-0.71	< 0.1	0.8322809	NR3C2	-0.14	-0.14	0.31	0.7402534
LSM5	-0.47	-0.47	ORF	0.7011449	PLD1	-0.14	-0.15	< 0.1	0.7580102
UBE2Q1	-0.43	-0.54	0.42	0.9229805	LRRC59	-0.14	-0.14	0.31	0.8393531
ZBTB10	-0.43	-0.47	0.27	0.9881244	DCUN1D3	-0.14	-0.23	0.42	0.7474078
MACROD2	-0.42	-0.42	< 0.1	0.9692401	MYCBP2	-0.13	-0.14	0.16	0.8707677
TRIM33	-0.42	-0.57	0.18	0.9819272	FAF1	-0.13	-0.32	0.31	0.8590793
GNG12	-0.42	-0.43	< 0.1	0.7042908	ZFP36L1	-0.13	-0.16	< 0.1	#N/D
NOVA1	-0.41	-0.41	0.3	0.9342027	AGPAT5	-0.12	-0.14	0.31	0.8383913
SH2D4A	-0.4	-0.42	< 0.1	0.851939	PIGO	-0.12	-0.12	0.31	0.723293
HMOX2	-0.39	-0.49	0.21	0.7894541	SON	-0.12	-0.12	0.3	0.7047923
ATXN7L1	-0.38	-0.38	0.17	0.7460288	SNX30	-0.11	-0.13	0.28	0.9268788
COL1A1	-0.37	-0.55	0.36	0.8815427	TNRC6B	-0.11	-0.15	0.28	0.9995629
ARGLU1	-0.36	-0.36	0.16	0.7713511	RPH3A	-0.11	-0.11	0.29	0.7705824
KCNMB2	-0.36	-0.36	< 0.1	0.7854116	SMTN	-0.11	-0.11	ORF	0.7733784
ZDHHC21	-0.36	-0.36	0.28	0.9910414	ATXN7L3B	-0.11	-0.24	0.26	0.8623476
CHP1	-0.36	-0.36	< 0.1	0.7753897	MMS19	-0.11	-0.19	0.16	0.7696984
TM6SF1	-0.35	-0.35	ORF	0.9977317	N4BP2L1	-0.1	-0.1	0.16	0.7476188
CRK	-0.35	-0.35	0.11	0.9431008	TERF2	-0.1	-0.38	0.28	0.9540062
LARP4	-0.34	-0.34	0.28	0.8933113	ZFHX4	-0.1	-0.1	0.16	0.8520022
VAV3	-0.34	-0.34	0.3	0.9359297	SPCS2	-0.1	-0.1	0.31	0.8454879
MAFB	-0.33	-0.34	0.28	0.9921495	STAG2	-0.1	-0.1	0.3	0.7948353
NUFIP2	-0.31	-0.4	0.28	0.9676238	PPP1R1A	-0.1	-0.12	0.31	0.7311639
LAS1L	-0.31	-0.31	ORF	0.772756	SOX6	-0.09	-0.27	0.48	0.9982879
IDE	-0.31	-0.4	0.28	0.8524781	ARMCX3	-0.09	-0.42	< 0.1	0.9973385
OTOF	-0.3	-0.3	< 0.1	0.8227932	PPP4R1	-0.09	-0.23	0.16	0.8466454
SLC27A4	-0.3	-0.3	< 0.1	0.8811743	RUNX2	-0.09	-0.09	< 0.1	0.7182284
TAF1	-0.29	-0.29	< 0.1	0.7831152	HERPUD2	-0.09	-0.53	0.43	0.9724498
ZDHHC18	-0.29	-0.29	0.3	0.8428726	DALRD3	-0.09	-0.09	ORF	#N/D
CACNB4	-0.29	-0.36	0.41	0.9562029	CADM2	-0.08	-0.08	0.31	0.7774932
LMO3	-0.28	-0.3	0.31	0.8672118	THSD7A	-0.08	-0.08	< 0.1	0.8739137
TPM3	-0.28	-0.29	0.31	0.9909119	NFIA	-0.08	-0.08	0.31	0.8318581
GNAQ	-0.28	-0.28	0.19	0.9962759	DUOX1	-0.08	-0.08	0.16	0.7466762
NOL4	-0.28	-0.28	0.13	0.8304263	FBXO33	-0.08	-0.08	0.16	0.7217258
KCND2	-0.28	-0.28	< 0.1	0.9987481	PKD2	-0.07	-0.07	0.16	0.762356
JMJD1C	-0.28	-0.28	< 0.1	0.7738174	MSI2	-0.07	-0.5	< 0.1	0.8661454
FGD1	-0.27	-0.27	0.16	0.7298374	LSM14A	-0.07	-0.11	0.3	0.7494714
MAP3K2	-0.27	-0.4	0.3	0.7912599	ATG9A	-0.07	-0.07	0.29	0.7307564
CELSR2	-0.26	-0.26	0.26	0.9993673	FRRS1L	-0.07	-0.07	0.27	0.8048913
PHF20	-0.26	-0.27	< 0.1	0.7955877	ABCA9	-0.07	-0.07	ORF	0.7806699
UPK1B	-0.25	-0.25	0.31	0.7491649	ZFAND3	-0.07	-0.11	0.31	0.7451599
PTPN12	-0.25	-0.25	0.31	0.9467405	TANC2	-0.07	-0.09	0.31	0.7704626
SALL1	-0.24	-0.37	0.52	0.9488729	DAB2IP	-0.06	-0.06	0.42	0.9180276
TBC1D8	-0.24	-0.31	0.16	0.8006183	SH3PXD2A	-0.06	-0.06	0.31	0.7403022
SRGAP3	-0.24	-0.43	0.28	0.9539175	MEF2C	-0.06	-0.28	0.39	0.9557015
SNAP29	-0.23	-0.65	< 0.1	0.8771082	MORC4	-0.05	-0.05	ORF	0.8936153

FAM120A	-0.23	-0.23	< 0.1	0.9196756	ATXN7L3	-0.05	-0.06	0.16	0.827499
AAK1	-0.22	-0.29	0.36	0.8111581	B4GALT7	-0.04	-0.71	< 0.1	0.9763423
EN1	-0.22	-0.22	0.3	0.7724608	ANKRD52	-0.04	-0.07	0.31	0.735103
RGS7BP	-0.22	-0.22	0.28	0.8376002	ATF2	-0.04	-0.06	0.16	0.7074413
ZBTB43	-0.22	-0.22	0.28	0.9916705	SHISA6	-0.04	-0.04	0.31	0.7072809
VAMP2	-0.22	-0.22	0.37	0.7005771	SERINC5	-0.04	-0.04	0.16	0.7677604
PTPLB	-0.22	-0.22	0.28	0.93297	ZBTB39	-0.04	-0.04	0.42	0.7347073
DUSP16	-0.22	-0.43	0.26	0.9944911	ESR2	-0.03	-0.03	< 0.1	0.817803
UBE2G1	-0.21	-0.21	0.31	0.8470159	ZBTB20	-0.03	-0.48	0.16	0.80438
UBLCP1	-0.21	-0.21	0.31	0.8034128	LHFPL4	-0.02	-0.38	0.52	0.894085
TMEM229B	-0.21	-0.21	0.31	0.7595688	MAP2	-0.02	-0.03	0.16	0.7113841
MANF	-0.21	-0.35	0.16	0.8511569	CD276	-0.02	-0.02	0.3	0.8733738
NEUROD1	-0.2	-0.2	0.16	0.7652747	USP7	-0.02	-0.13	0.31	0.9645856
MTDH	-0.2	-0.2	0.31	0.7470866	GATA6	-0.02	-0.15	0.31	0.9014495
HIVEP2	-0.2	-0.2	0.3	0.7563388	ARHGAP32	-0.02	-0.04	0.31	0.7903784
FBXW7	-0.2	-0.2	0.16	0.8028841	DNMT3A	-0.02	-0.1	0.35	0.8108802
RAB14	-0.19	-0.43	0.31	0.8961808	F11R	-0.01	-0.29	0.31	0.8308495
CACNA2D1	-0.18	-0.19	< 0.1	0.9027537	RNF217	-0.01	-0.11	0.31	0.7284629
ARL5A	-0.18	-0.18	0.31	0.8389501	MSL2	-0.01	-0.14	0.16	0.7433192
CAMK2G	-0.18	-0.18	0.31	0.8574712	SLC16A10	-0.01	-0.23	0.24	0.7546558
HAPLN1	-0.17	-0.17	0.4	0.7415332	HNRNPL	0	-0.56	0.25	0.9357574
MAF	-0.17	-0.17	0.23	0.7829193	FGF10	0	-0.26	0.16	0.7599578
NKX2-1	-0.17	-0.21	0.31	0.716263	COLEC10	0	-0.25	0.16	0.8082429
CAMK2A	-0.17	-0.17	0.31	0.9495654					

ORF, Open Reading Frame
