# Supplementary material for: Downregulation of circulating miR 802‐5p and miR 194‐5p and upregulation of brain MEF2C along breast cancer brain metastasization
Source: Mol Oncol. 2020 Feb 5;14(3):520–38. doi: 10.1002/1878-0261.12632 (PMC7053247; doi:10.1002/1878-0261.12632)
Supplement: Supplementary file 8 — Table S8. Results of the target prediction for miR‐205‐5p using TargetScan v.7.2. and diana tools MicroT‐CDS v.5.0. [file MOL2-14-520-s008.pdf]

Supplementary Table 8. Results of the target prediction miR-205-5p using TargetScan v.7.2. and DIANA Tools MicroT-CDS v.5.0.

Target Gene	Cumulative weighted context++ score	Total context++ score	Aggregate PCT	MiTG	Target Gene	Cumulative weighted context++ score	Total context++ score	Aggregate PCT	MiTG
CHN1	-0.92	-0.92	0.97	0.999993252	RBFOX3	-0.15	-0.15	0.17	0.73603808
MTFR1L	-0.73	-0.76	< 0.1	0.999525229	ERBB4	-0.15	-0.19	0.84	0.98597218
RBMX	-0.67	-0.68	< 0.1	0.952901649	LPCAT1	-0.15	-0.23	0.6	0.975751437
SH3GL3	-0.63	-0.63	ORF	0.733970037	SLC24A2	-0.15	-0.15	0.17	0.778414112
CDH11	-0.6	-0.66	0.25	0.981799127	MAGI2	-0.15	-0.15	0.55	0.920241676
SLC35B3	-0.58	-0.66	0.87	0.938354779	RUNX2	-0.15	-0.15	0.51	0.848211814
RNF146	-0.58	-0.58	ORF	0.72278313	FAM104A	-0.14	-0.27	< 0.1	0.754973635
COL16A1	-0.56	-0.56	0.6	0.926933374	CPEB2	-0.14	-0.14	0.56	0.934905009
HS3ST1	-0.54	-0.55	0.89	0.99546152	PPP3R1	-0.14	-0.14	0.15	0.914369043
SEPT4	-0.49	-0.49	ORF	0.99127931	MED1	-0.14	-0.15	0.12	0.979514007
SLC35A1	-0.49	-0.49	0.38	0.991811429	ARFGEF1	-0.14	-0.18	0.16	0.84797578
GXYLT1	-0.47	-0.47	0.45	0.914210714	USP7	-0.14	-0.15	0.2	0.857230071
RCBTB1	-0.47	-0.51	0.18	0.997861681	STK3	-0.14	-0.14	0.15	0.717986453
SSBP3	-0.46	-0.46	0.8	0.919610945	CDC27	-0.13	-0.2	0.55	0.961094045
SELT	-0.45	-0.45	0.74	0.919103634	CREBRF	-0.13	-0.13	0.34	0.825448902
BICC1	-0.43	-0.43	0.58	0.984277347	AMOT	-0.13	-0.18	0.25	0.87796691
MARCKS	-0.43	-0.45	0.94	0.992188679	DOK4	-0.13	-0.24	0.4	0.99787169
NDUFA4	-0.43	-0.44	0.63	0.885879847	TNRC6C	-0.12	-0.13	0.43	0.704901862
ACSL1	-0.43	-0.49	0.54	0.999853733	RAB11FIP1	-0.12	-0.33	0.77	0.87174434
LENG9	-0.43	-0.43	< 0.1	0.738991679	GRB10	-0.12	-0.15	0.15	0.734196062
ARRB2	-0.42	-0.42	ORF	0.720210716	UBIAD1	-0.12	-0.25	0.23	0.905361141
RAP2B	-0.41	-0.6	0.72	0.751604115	ZCCHC14	-0.11	-0.12	0.82	0.999888663
CASC4	-0.39	-0.39	< 0.1	0.999057233	YES1	-0.11	-0.12	0.19	0.776862968
CTPS2	-0.38	-0.54	0.27	0.966235919	BCL6	-0.11	-0.11	0.15	0.801774655
RTN3	-0.34	-0.34	ORF	0.979534977	PJA2	-0.11	-0.24	< 0.1	0.706322003
DDX5	-0.34	-0.34	0.36	0.990647314	NAA10	-0.11	-0.11	ORF	0.712775437
PTPRM	-0.34	-0.34	0.48	0.980408592	FAM84B	-0.11	-0.22	0.64	0.963696843
LHFPL2	-0.33	-0.33	0.13	0.934212645	MICAL2	-0.11	-0.11	0.41	0.992118624
FAM126A	-0.33	-0.43	0.32	0.97998278	LCOR	-0.1	-0.1	0.49	0.938548892
SGMS1	-0.32	-0.33	0.28	0.88687212	GTF3C2	-0.1	-0.16	0.14	0.832298378
UBE2N	-0.32	-0.35	0.55	0.916768162	LYSMD4	-0.1	-0.1	0.16	0.880004607
NAA11	-0.32	-0.32	< 0.1	0.802985979	SMIM14	-0.1	-0.1	0.16	0.803350247
CRMP1	-0.32	-0.32	0.15	0.845437419	UBE2E3	-0.1	-0.12	0.15	0.900154621
EZR	-0.31	-0.4	0.18	0.877699421	ITGA5	-0.09	-0.09	0.15	0.870049795
TMEM255A	-0.31	-0.31	0.37	0.966052396	DNM3	-0.09	-0.18	0.15	0.941321691
SBF2	-0.31	-0.34	0.74	0.998177791	AP1G1	-0.09	-0.09	0.14	0.896858213
LSAMP	-0.31	-0.52	0.24	0.970999734	PPP1R3A	-0.09	-0.09	0.15	0.885846296
ABHD17B	-0.3	-0.3	0.2	0.987641095	UBFD1	-0.09	-0.09	0.15	0.847038958
ADAMTS9	-0.3	-0.3	0.82	0.999292073	STRBP	-0.09	-0.09	0.15	0.94855762
NTNG1	-0.29	-0.29	< 0.1	0.985800072	CSF1	-0.09	-0.09	0.52	0.86669592
AAK1	-0.29	-0.3	0.79	0.929579165	KLHL31	-0.09	-0.14	< 0.1	0.733184382
MFNG	-0.28	-0.28	0.13	0.888952325	KLF7	-0.09	-0.13	0.24	0.870669959
ZEB2	-0.27	-0.31	0.2	0.949596923	ACBD5	-0.09	-0.09	0.15	0.740118816
CALU	-0.27	-0.43	0.48	0.926005913	RBPMS2	-0.09	-0.41	0.27	0.847065105
PHC2	-0.27	-0.27	0.59	0.999909005	AXIN2	-0.09	-0.09	0.15	0.811660502
LIMS2	-0.26	-0.26	0.21	0.882267543	HMGB1	-0.08	-0.23	0.16	0.766985227
GATA3	-0.26	-0.36	0.44	0.8607067	STXBP6	-0.08	-0.26	< 0.1	0.768967766
RAB11FIP2	-0.26	-0.27	0.17	0.779180309	SCMH1	-0.08	-0.08	0.46	0.722155904
ELF1	-0.26	-0.27	0.16	0.932311147	ETF1	-0.08	-0.09	0.19	0.892559419
FBXO24	-0.26	-0.26	< 0.1	0.963043192	LRCH3	-0.08	-0.34	0.65	0.999225786
RBM47	-0.25	-0.25	0.37	0.96075938	GALNT3	-0.08	-0.08	< 0.1	0.797236432
ENC1	-0.25	-0.26	0.54	0.933483908	PAFAH1B1	-0.08	-0.13	< 0.1	0.709441751
DGCR8	-0.25	-0.25	0.15	0.792630328	ZEB1	-0.08	-0.08	0.59	0.95658008
PHB	-0.24	-0.24	0.27	0.942033265	KLHL15	-0.08	-0.08	0.34	0.919104238
ESRRG	-0.24	-0.24	0.6	0.944665844	KAZN	-0.07	-0.21	0.16	0.704917971
MGRN1	-0.24	-0.24	0.91	0.960333878	PTPRJ	-0.07	-0.08	0.52	0.864906774
CBX1	-0.23	-0.23	0.3	0.865406798	SRGAP1	-0.07	-0.1	0.13	0.910910396

DUSP7	-0.23	-0.39	0.84	0.990130059
CNIH1	-0.23	-0.24	< 0.1	0.727955829
IRF2BPL	-0.23	-0.23	0.15	0.847668051
FNBP1L	-0.23	-0.23	0.15	0.857705505
TBCEL	-0.23	-0.4	0.22	0.882258541
MLLT4	-0.23	-0.23	0.74	0.994133999
EAF1	-0.23	-0.24	< 0.1	0.810976238
SLC19A2	-0.23	-0.23	0.18	0.862316937
VEGFA	-0.22	-0.23	0.53	0.964016185
MIER3	-0.22	-0.25	0.15	0.794972754
MMD	-0.22	-0.22	< 0.1	0.866612867
DLG2	-0.22	-0.22	0.6	0.983278957
FA2H	-0.22	-0.22	0.29	0.803072096
ERRFI1	-0.22	-0.24	0.2	0.965187168
HIATL1	-0.22	-0.22	< 0.1	0.751577033
E2F1	-0.21	-0.21	0.19	0.772707982
CCDC43	-0.21	-0.21	0.17	0.892674628
IL1R1	-0.21	-0.21	0.13	0.888622728
RBMS1	-0.21	-0.21	0.59	0.922485683
RNF157	-0.21	-0.23	0.46	0.871539997
CCDC176	-0.21	-0.21	< 0.1	0.838593893
NACC2	-0.21	-0.3	0.97	0.998641047
CLK3	-0.2	-0.2	0.13	0.819476251
PLCB1	-0.2	-0.2	0.15	0.999110121
FAM155A	-0.2	-0.2	0.32	0.954006614
FAM168A	-0.2	-0.29	0.31	0.835083967
RORA	-0.19	-0.23	0.57	0.993529416
TNR	-0.19	-0.19	0.35	0.831282215
NCOA1	-0.19	-0.2	0.29	0.804713828
CALCRL	-0.19	-0.51	0.86	0.837944438
GLIS3	-0.18	-0.18	0.45	0.920216282
TRAK2	-0.18	-0.18	0.26	0.972603855
BAMBI	-0.18	-0.18	0.16	0.944987005
AFF1	-0.18	-0.3	0.15	0.763660049
UBE2G1	-0.18	-0.21	0.12	0.711123012
FRK	-0.18	-0.43	0.98	0.992187598
PAX9	-0.18	-0.21	0.38	0.959988208
TMEM236	-0.18	-0.18	0.33	0.8062065
NFIB	-0.18	-0.2	0.56	0.963019587
TMEM236	-0.17	-0.17	0.34	0.8062065
HERC3	-0.17	-0.21	0.23	0.871832487
VTI1B	-0.17	-0.2	0.15	0.760560931
TXNRD1	-0.16	-0.16	0.16	0.714739043
TLK1	-0.16	-0.2	0.29	0.762889503
TM9SF3	-0.16	-0.2	0.36	0.877885712
RAB9B	-0.15	-0.15	0.32	0.772182941
TTI1	-0.15	-0.16	0.15	0.762503424
LUC7L3	-0.07	-0.25	< 0.1	0.773759121
KSR1	-0.07	-0.07	0.6	0.754976653
NFAT5	-0.07	-0.09	0.79	0.888654354
DSC1	-0.07	-0.07	ORF	0.816335742
POU2F1	-0.06	-0.07	0.45	0.988942977
WWC2	-0.06	-0.07	0.39	0.746953504
CCDC93	-0.06	-0.21	0.16	0.750168736
HNRNPK	-0.06	-0.06	0.16	0.780812016
TNRC6B	-0.05	-0.07	0.15	0.793505058
NHS	-0.05	-0.05	< 0.1	0.792377623
PRKCA	-0.05	-0.09	< 0.1	0.872703581
DDX52	-0.05	-0.09	0.16	0.864676578
TRPS1	-0.05	-0.06	0.18	0.724851769
LIN9	-0.04	-0.23	0.53	0.955846215
ACTB	-0.04	-0.16	< 0.1	0.727822925
MTRF1L	-0.04	-0.04	ORF	0.72165992
KMT2A	-0.04	-0.04	0.39	0.988582169
CMTM4	-0.04	-0.12	0.15	0.796109514
MAGI1	-0.03	-0.08	0.2	0.715465315
PSD3	-0.03	-0.1	0.16	0.949168421
TIAL1	-0.03	-0.03	0.16	0.802738327
CDC42BPB	-0.03	-0.03	< 0.1	0.826726593
SEPT3	-0.03	-0.03	< 0.1	0.742390638
RARA	-0.03	-0.1	0.15	0.726973094
MTMR4	-0.03	-0.03	< 0.1	0.861373076
MAP3K9	-0.03	-0.03	0.15	0.801937473
STK10	-0.03	-0.29	0.49	0.846486873
RPS6KA3	-0.03	-0.17	0.61	0.906383808
NSUN5	-0.03	-0.15	0.16	0.939357455
HOXD13	-0.02	-0.21	0.23	0.96584158
PDE7A	-0.02	-0.02	0.16	0.940721633
ARMC8	-0.02	-0.12	< 0.1	0.914997176
MPRIP	-0.02	-0.02	< 0.1	0.920842137
NR3C2	-0.01	-0.01	0.15	0.812906919
IPPK	-0.01	-0.19	0.39	0.893529093
ANK2	-0.01	-0.01	ORF	0.858822353
LRP6	-0.01	-0.01	0.16	0.77409024
ARID1A	-0.01	-0.01	0.15	0.72157762
TMEM136	0	-0.14	0.17	0.766204693
PAPD5	0	-0.09	0.84	0.987748336
SIPA1L1	0	-0.15	0.74	0.939530097
EIF4E	0	-0.12	0.16	0.745426026
TM9SF2	0	-0.28	0.29	0.975710769
CADM1	0	-0.23	0.8	0.998396394
ZBTB20	0	-0.38	0.9	0.999816235
EPS8	0	-0.1	0.18	0.814913423

ORF, Open Reading Frame
