# Supplementary material for: Downregulation of circulating miR 802‐5p and miR 194‐5p and upregulation of brain MEF2C along breast cancer brain metastasization
Source: Mol Oncol. 2020 Feb 5;14(3):520–38. doi: 10.1002/1878-0261.12632 (PMC7053247; doi:10.1002/1878-0261.12632)
Supplement: Supplementary file 9 — Table S9. Results of the target prediction for miR‐92a‐1‐5p using TargetScan v.7.2 and Diana MicroT‐CDS v.5.0. [file MOL2-14-520-s009.pdf]

Supplementary Table 9. Results of the target prediction for miR-92a-1-5p using TargetScan v.7.2 and Diana MicroT-CDS v.5.0.

Target Gene	Cumulative weighted context++ score	Total context++ score	Aggregate PCT	MiTG	Target Gene	Cumulative weighted context++ score	Total context++ score	Aggregate PCT	MiTG
SLC25A23	-1.27	-1.27	N/A	0.724338153	RPH3AL	-0.19	-0.28	N/A	0.719469008
ANAPC15	-0.75	-0.75	N/A	0.740113401	ENG	-0.18	-0.18	N/A	0.711306376
SF3A3	-0.67	-0.67	N/A	0.765194043	ZBTB16	-0.18	-0.2	N/A	0.704966765
YBX3	-0.58	-0.59	N/A	0.91528158	NFIC	-0.17	-0.17	N/A	0.897584357
PAX5	-0.55	-0.55	N/A	0.73296523	PLEC	-0.17	-0.17	N/A	0.805138175
FOXP1	-0.54	-0.54	N/A	0.74974079	DUSP13	-0.17	-0.17	N/A	0.725291403
UBQLN4	-0.54	-0.54	N/A	0.73940766	PVR	-0.16	-0.16	N/A	0.804009942
PPME1	-0.5	-0.5	N/A	0.993161162	MATN1	-0.16	-0.16	N/A	0.702411274
CEP128	-0.47	-0.47	N/A	0.710240527	ANO5	-0.15	-0.2	N/A	0.901922797
MSI1	-0.46	-0.46	N/A	0.883522177	CNTN4	-0.15	-0.16	N/A	0.793381793
NANP	-0.45	-0.45	N/A	0.765256616	QSER1	-0.15	-0.18	N/A	0.725760411
WNK3	-0.44	-0.44	N/A	0.906745437	SEMA5A	-0.14	-0.14	N/A	0.917690472
FBXL16	-0.41	-0.41	N/A	0.897573645	PLEKHO2	-0.14	-0.14	N/A	0.819790157
NECAB1	-0.4	-0.4	N/A	0.867738479	WASF2	-0.14	-0.14	N/A	0.723182919
NECAB1	-0.4	-0.4	N/A	0.797531498	SEPT11	-0.13	-0.13	N/A	0.822719738
PRPF3	-0.39	-0.39	N/A	0.741453403	UGGT1	-0.11	-0.32	N/A	0.731965789
KCNC1	-0.39	-0.39	N/A	0.704196136	CLSTN2	-0.1	-0.18	N/A	0.934204647
ENSA	-0.34	-0.43	N/A	0.771236868	TNPO1	-0.1	-0.1	N/A	0.96483842
AAK1	-0.31	-0.39	N/A	0.732052895	DNAJC5	-0.1	-0.12	N/A	0.847838553
DOC2A	-0.3	-0.3	N/A	0.828597686	SLC6A17	-0.1	-0.1	N/A	0.725119025
TRPS1	-0.3	-0.3	N/A	0.709692094	PAPPA	-0.09	-0.09	N/A	0.735435294
FBXW7	-0.29	-0.29	N/A	0.839990156	ANGEL1	-0.07	-0.07	N/A	0.844329569
DCLK2	-0.29	-0.29	N/A	0.744061585	MLX	-0.07	-0.26	N/A	0.742852151
ELK1	-0.28	-0.28	N/A	0.871306395	ARPP19	-0.06	-0.18	N/A	0.886682773
SERPINA6	-0.28	-0.28	N/A	0.716462407	EIF4E3	-0.05	-0.18	N/A	0.810498788
TMEM167B	-0.26	-0.26	N/A	0.784646285	TNRC6B	-0.04	-0.04	N/A	0.744628843
LRRN2	-0.26	-0.26	N/A	0.774779473	NOS1	-0.03	-0.03	N/A	0.816510378
BACH2	-0.26	-0.27	N/A	0.75160741	WIPF2	-0.03	-0.16	N/A	0.810571013
SV2B	-0.25	-0.35	N/A	0.822705169	LPHN1	-0.03	-0.05	N/A	0.735511748
EPB42	-0.25	-0.25	N/A	0.746322714	HNF1A	-0.02	-0.02	N/A	0.915450583
JPH4	-0.24	-0.24	N/A	0.759273395	GAS7	-0.01	-0.16	N/A	0.830489739
NAV1	-0.23	-0.29	N/A	0.807522062	LIG3	-0.01	-0.17	N/A	0.713390023
ZBTB7A	-0.22	-0.22	N/A	0.727907619	SLC22A1	0	-0.14	N/A	0.902476233
ATP2B4	-0.22	-0.22	N/A	0.709972891	SLC16A3	0	-0.18	N/A	0.891252288
BSDC1	-0.21	-0.21	N/A	0.794279074	ZBTB20	0	-0.05	N/A	0.868198765
MMP9	-0.21	-0.42	N/A	0.706774012	ERCC6	0	-0.01	N/A	0.78574321
NTNG1	-0.2	-0.2	N/A	0.812611243	SRPK2	0	-0.41	N/A	0.778547884
POP5	-0.2	-0.2	N/A	0.758119971					

N/A, Not applicable
