# Supplementary material for: Downregulation of circulating miR 802‐5p and miR 194‐5p and upregulation of brain MEF2C along breast cancer brain metastasization
Source: Mol Oncol. 2020 Feb 5;14(3):520–38. doi: 10.1002/1878-0261.12632 (PMC7053247; doi:10.1002/1878-0261.12632)
Supplement: Supplementary file 10 — Table S10. Results of the target prediction for miR‐181a‐3p using TargetScan v.7.2. and diana tools MicroT‐CDS v.5.0. [file MOL2-14-520-s010.pdf]

Supplementary Table 10. Results of the target prediction for miR-181a-3p using TargetScan v.7.2. and DIANA Tools MicroT-CDS v.5.0.

Target Gene	Cumulative weighted context++ score	Total context++ score	Aggregate PCT	MiTG	Target Gene	Cumulative weighted context++ score	Total context++ score	Aggregate PCT	MiTG
XIRP2	-0.68	-0.68	N/A	0.728971265					

N/A, Not Applicable
